# Role of hydration energy and co-ions association on monovalent and divalent cations adsorption at mica-aqueous interface

**DOI:** 10.1038/s41598-018-30549-9

**Published:** 2018-08-15

**Authors:** Sai Adapa, Ateeque Malani

**Affiliations:** Department of Chemical Engineering, Indian Institute of Technology Bombay, Mumbai, MH 400076 India

## Abstract

Adsorption of ions at the solid - aqueous interface is the primary mechanism in fast biological processes to very slow geological transformations. Despite, little is known about role of ion charge, hydration energy and hydration structure on competitive adsorption of ions, their structure and coverage at the interface. In this report, we investigate the structure and adsorption behavior of monovalent (Rb^+^ and Na^+^) and divalent (Sr^2+^ and Mg^2+^) cations ranging from 0–4.5 M of bulk concentrations on the muscovite mica surface. Divalent ions have stronger adsorption strength compared to monovalent ions due higher charge. However, we observed counter-intuitive behavior of lesser adsorption of divalent cations compared to monovalent cations. Our detailed analysis reveals that hydration structure of divalent cations hinders their adsorption. Both, Na^+^ and Rb^+^ ions exhibits similar adsorption behavior, however, the adsorption mechanism of Na^+^ ions is different from Rb^+^ ions in terms of redistribution of the water molecules in their hydration shell. In addition, we observed surface mediated RbCl salting out behavior, which is absent in Na^+^ and divalent ions. We observed direct correlation in hydration energy of cations and their adsorption behavior. The obtained understanding will have tremendous impact in super-capacitors, nanotribology, colloidal chemistry and water purifications.

## Introduction

Adsorption of ions on the charge surface is a fundamental approach widely used in remediation of heavy metals^1–3^, water purification^4–7^, minerals formation^8^, surface-mediated reactions^9,10^, manufacturing of molecular electronic devices^11^ and energy storage devices like super-capacitors^12^. In these processes, distribution of counter- and co-ions from the surface to the bulk region creates an electrical double layer (EDL) at the interface^13^. In EDL, some of the ions are in direct association with the surface (known as the Stern layer) while the rest of the ions are distributed away from the surface till the bulk region (known as the diffuse layer)^14,15^. The Gouy-Chapman theory assumes ions to be point charges and describe their distribution in the diffuse layer successfully, however, fails to predict their adsorption in the Stern layer^16,17^. Thus, a molecular level understanding of the ions distribution, role of hydration energy and co-ions association in the Stern layer is essential for enhancing the above processes, especially for improving charge densities in electrochemical devices^18,19^ to meet the growing energy demand, and for exploiting the metal ion exchange capability of naturally available clay minerals for remediation of nuclear waste^1,20,21^.

In this report, we have studied the adsorption behavior of monovalent (Rubidium - Rb^+^ and Sodium - Na^+^) and divalent (Magnesium - Mg^2+^ and Strontium - Sr^2+^) cations at mineral surface highlighting the importance of the ion charge, hydration energy and hydration structure. Since divalent ions have higher electrostatic interactions, we expected their adsorption to be higher as compared to monovalent ions. However, we observed a counter-intuitive behavior of lesser adsorption of divalent ions at all concentrations as compared to monovalent ions. We have analyzed the energetics, structure, and clusters of adsorbed ions to explain observed behavior.

Majority of studies focused on understanding the properties of ions at solid - aqueous interface have used muscovite mica surfaces in their work. The reasons are that (a) muscovite mica surfaces are easy to cleave, (b) produces atomically smooth surfaces,^22^ (c) the framework carries a negative charge compensated by surface potassium ions (K^+^), and (d) these surface (K^+^) ions can be exchanged with other cations in the presence of aqueous salt solutions^23–26^. Experimental studies using surface force apparatus (SFA) on electrolyte solutions between two mica surfaces have observed short-range repulsive forces due to dehydration of ions beyond a critical concentration of salt solution^27^. Further, this critical concentration was found to vary with the type of ions suggesting that their individual hydration structure and energy plays the significant role in ion adsorption at surfaces. The X-ray reflectivity (XRR) studies of mica - water system established that water molecules and surface cations are adsorbed on the ditrigonal cavities of mica surface^28^. In addition, non-uniform fluctuations in the density of the interfacial water layer were observed up to 10 Å from the mica surface^28^. In subsequent studies, it was found that Rb^+^ ions are adsorbed in partially hydrated states, whereas Sr^2+^ ions are observed in both, partially and fully hydrated states within the Stern layer^29^. The adsorption location of ions in the lateral and normal direction obtained from AFM studies of electrolyte solution on the mica surface are in concurrence with the XRR studies^29–32^. The recent atomic force microscopy (AFM) studies found that Rb^+^ ions adsorb together forming a cluster on mica surface, driven by the energetics of adsorbing ions and confirmed using molecular dynamics (MD) simulations^30^. AFM studies of KCl solution at higher concentration have found signatures of crystal formation at the mica surface^31,33^. Unfortunately, the SFA and AFM studies are restricted to the dilute concentrations due to the possibility of disturbing the Stern layer. Measurements from the XRR technique provides structural information of interfacial water and ions perpendicular to the surface alone and does not provide in-plane behavior^23^. Recent surface X-ray diffraction (SXRD) studies have found co-ions as well in the Stern layer^23,26^. Recent AFM techniques on the mica-RbCl solution (0–4 M) are able to probe in the three dimensional structures of solutions on the surface but still has the limitation to distinguish ion hydration and free water molecules^31^.

Molecular simulations have served as an important tool for providing a molecular-level understanding of the experimental observations. MD simulations have shown that layering of water molecules adjacent to the mica surface is due to hydration of surface cations^34–37^. Water molecules of first contact layer adsorbs on ditrigonal cavities, whereas the second layer hydrate the cations^35^. The orientation, hydrogen bonding, dynamic relaxation and viscosity of water molecules in these layers is different compared to bulk water due to the direct and indirect effect of mica surface^34–36,38,39^. MD simulation studies of Li^+^, Na^+^, K^+^, Rb^+^ and Cs^+^ ions at dilute concentration showed that these ions adsorb in the inner sphere complexes which agrees with XRR results^36,39^. Further, the adsorption free energy of these ions near the surface depends on the ability of the mica surface to satisfy their hydration structure^39,40^. The adsorption energy of divalent ions was found to be much higher than compared to monovalent ions^40^. Previous experimental and computational studies have established the location of ion adsorption, their energy and presence of co-ions near the surface. Despite these studies, the complete picture about structure of adsorbed ions, their hydration shell and association with co-ions at different concentration remains elusive.

We hypothesize that, the co-operative effect of counter-ions, competitiveness between counter-ions and charge balancing ions (K^+^ in case of mica surface), and the ion-pairing effect of co-ions (cation-anion) would be significant along with surface hydration, especially at higher aqueous electrolyte concentration near charged solid substrate. These effects would significantly change the structure of adsorbed ions and water molecules, hydration of cations and may lead to cation-anion cluster formation. In this work, MD simulations of aqueous solutions at various concentrations from 0–4.5 M were performed to check our hypothesis. In order to understand the effect of ion charge and hydration energy, we considered aqueous chloride (Cl^−^) solution of monovalent (Rb^+^ and Na^+^) and divalent (Sr^2+^ and Mg^2+^) cations on the mica surface. We chose Rb^+^ ions, as its hydration energy is comparable to mica surface K^+^ ions, whereas, Na^+^ ions have higher hydration energy (Table 1). The Mg^2+^ and Sr^2+^ ions have higher hydration energy (~5 times) than K^+^ ion. We observed counter-intuitive adsorption behavior of lesser adsorption of divalent ions at all concentrations as compared to monovalent ions. We explained the reason using energetics and structural analysis. While adsorption isotherms of monovalent (Rb^+^ and Na^+^) ions exhibit similar behavior, however a distinct mechanism of hydration water redistribution and association with Cl^−^ ions is observed. To the best of our knowledge, this is the first work which studies extensively the salt solutions of multiple ions near solid surface for large concentration range (0–4.5 M) capturing various adsorption regimes and providing molecular details. This information will aid in various areas of application such as super-capacitors, nanotribology, colloidal chemistry, heavy metal removals, and geochemistry.

**Table 1:** Adsorption free energies (Δ*A*) of cations (K^+^, Na^+^, Rb^+^, Mg^2+^ and Sr^2+^) and water molecule (Ow) at the mica - water interface, comparison with literature XRR study (Δ*G*)^60^ and hydration energy of ions in bulk (Δ*G*_*hyd*_)^61^.

Cations	Δ*A*, kcal mol^−1^		Δ*G* (Exp.) kcal mol^−1^	Δ*G*_*hyd*_ kcal mol^−1^
	AS_1_	AS_2_		
K^+^	−7.85 (1.80 Å)		−5.31	−70.51
Rb^+^	−7.70 (1.85 Å)		−5.61	−65.73
Na^+^	−6.70 (1.46 Å)	−2.43 (4.15 Å)	−3.42	−87.24
Sr^2+^	−6.03 (2.56 Å)	−4.19 (4.18 Å)		−329.83
Mg^2+^	−15.67 (2.31 Å)	−11.08 (3.63 Å)		−437.38
Water	−1.81 (1.85 Å)	−1.20 (2.75 Å)		

Numbers in brackets are the location of free energy minimum. AS_1_ - Adsorption state 1, AS_2_ - Adsorption state 2.

## Results and Discussion

### Adsorption isotherms

**Figure 1:**
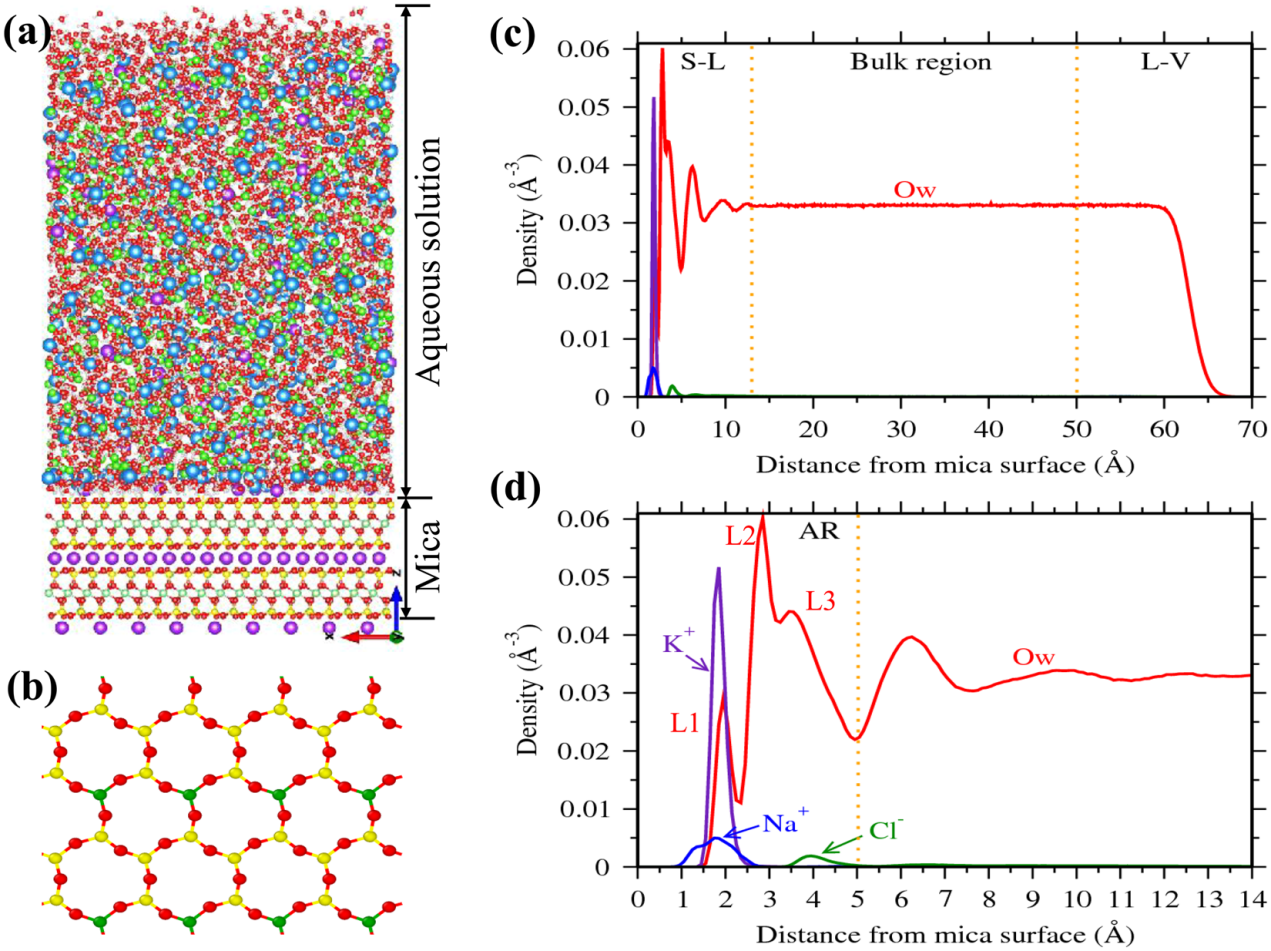
Representative simulation cell, mica surface and density distribution near the surface. **(a)** Simulation cell consisting of aqueous salt solution near muscovite mica surface (Oxygen - red, Hydrogen - white, Cation - blue, Chlorine - green, Silicon - yellow, Potassium - violet, Aluminum(of mica) - green). **(b)** Top view of mica surface (001 plane) showing ditrigonal cavities (often referred to as hexagonal cavities, formed by silicon, aluminum and bridging oxygen atoms) without surface potassium (K^+^) ions. **(c)** and **(d)** Representative density profiles of cations and water (oxygen, Ow) normal to the mica surface. The reference, i.e. *z* = 0, corresponds to the plane passing through basal bridging oxygens of mica surface. Space above mica surface is divided into interfacial (0–13 Å), bulk (13–50 Å) and liquid-vapor (≥50 Å) regions based on water density profile (indicated by vertical dotted lines). **(d)** Closer view of density profile of the interfacial region, where profound oscillations in water density is observed. In water density profile, first peak (L1) represents the adsorbed water molecules on the cavities and L2 + L3 are the water molecules hydrating surface ions and/or forming hydrogen bonds with either surface oxygens or with other water molecules. Ions (K^+^ - violet, Na^+^ - blue and Cl^−^ - green) and water molecules (Ow - red) adsorbed between 0–5 Å (referred to as adsorption region, AR) are in direct association with the surface.

A representative density profile of water and ions adjacent to the mica surface is shown in Fig. 1. The ions are found to be adsorbed mostly around 2–3 Å from the mica surface. These adsorbed ions directly interact with the mica surface by forming a hydration shell with the surface oxygen atoms (Fig. 1a). In case of water molecules, we observed large oscillation in the density profile near the mica surface due to the presence of surface ions (Fig. 1c). The locations and extend of these oscillations are consistent with the experimental studies^28,32^, as shown in our previous work^35^. Within the 13 Å of the interfacial region, density oscillations are more pronounce between 0–5 Å region (Fig. 1d). Our previous studies have shown that these density oscillations are due to hydration of surface ions by the interfacial water molecules^35,38^. In this study, we considered this 0–5 Å region as the adsorption region (AR) and the concentration of ions in this AR (C_*A*_) is considered as adsorbate loading. We further observed that water density in the 13–50 Å region is constant and corresponds to the bulk density of water; the ion concentration within this region is defined as bulk concentration (C_*B*_).

**Figure 2:**
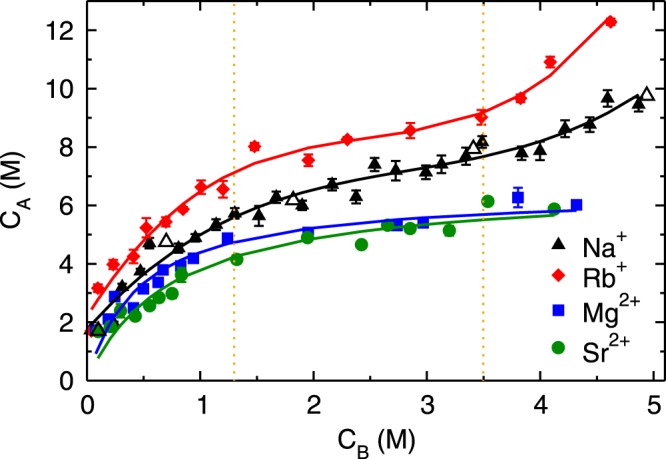
Adsorption isotherms of cations. Adsorption isotherms (cation concentration in AR, C_*A*_ vs. bulk region, C_*B*_) of sodium (Na^+^ - ▲), rubidium (Rb^+^ - ♦), magnesium (Mg^2+^ - ■) and strontium (Sr^2+^ - •) ions on the mica surface. The isotherms of monovalent (Rb^+^ and Na^+^) ions exhibits three regimes, whereas only two regimes are observed for divalent (Mg^2+^ and Sr^2+^) ions. Open symbol corresponds to adsorption data for larger NaCl system (containing *N*_*w*_ ≈ 8500 water molecules), which are in agreement with data of smaller system sizes (*N*_*w*_ ≈ 5000).

Figure 2 shows the adsorption isotherms (C_*A*_ vs. C_*B*_) of monovalent (Rb^+^ and Na^+^) and divalent (Mg^2+^ and Sr^2+^) cations obtained by performing MD simulation of aqueous solutions at various C_*B*_. The adsorption isotherms of both monovalent ions exhibit three regimes; (a) an initial rapid increase for C_*B*_ < 1.3 M followed by (b) linear rise between 1.3 ≤ C_*B*_ < 3.5 M and (c) subsequent exponential growth beyond C_*B*_ ≥ 3.5 M (henceforth referred to as first, second and third regime, respectively). The adsorption quantity of Rb^+^ ions is higher compared to Na^+^ ions at all C_*B*_, nevertheless, the range of three regimes is similar in both of the monovalent ions. Such characteristic is classified as type-II behavior in the International Union of Pure and Applied Chemistry (IUPAC) classification^41^, which is generally attributed to the multilayer adsorption, as observed for water adsorption on mica surface^42^. The divalent ions exhibit an early increase in adsorption quantity followed by saturation, which is characterized as type-I behavior in IUPAC classification^41^, and attributed to the mono-layer adsorption. The adsorption quantity of divalent ions is lesser as compared to monovalent ions at all C_*B*_, which is a counter-intuitive observation. As the divalent ions have higher charge and hence electrostatic interactions as compared to monovalent ions, we expected their adsorption to be higher than monovalent ions. We also observed that the difference in adsorption behavior of simple and heavy metal ions (Na^+^ vs. Rb^+^ and Mg^2+^ vs. Sr^2+^) is non-significant. In order to understand the observed variation in adsorption behavior, we have investigated the energetics and hydration structure of adsorbed ions near the mica surface as described below. We first describe the adsorption behavior of monovalent ions followed by divalent ions.

### Free Energy Profile of Adsorbed Ions

**Figure 3:**
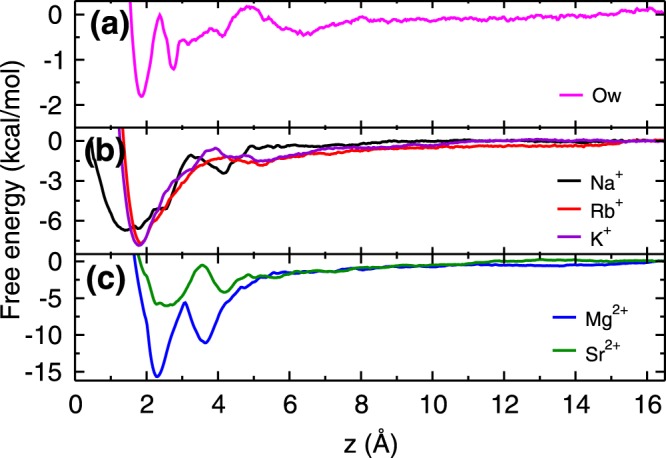
Energetics of Adsorption. Free energy profiles of (**a**) water molecule, (**b**) monovalent ions and (**c**) divalent ions at the mica - water interface. In **(a)**, first minimum corresponds to the water adsorbed in ditrigonal cavities and rest are due to hydration of surface K^+^ ions. All ions have attractive free energy minima (of varying strength) near the mica surface. While Rb^+^ and K^+^ ions have single minimum, a broader free energy distribution is observed for Na^+^ ions. Both (Mg^2+^ and Sr^2+^) divalent ions have two adsorption minima near mica surface within the AR.

Figure 3 shows free energy profiles of cations (K^+^, Na^+^, Rb^+^, Mg^2+^ and Sr^2+^) in water adjacent to the mica surface, calculated using the umbrella sampling technique^43,44^ and the weighted histogram analysis method^45^ (details are given in section 1 of Supplementary Information (SI)). The obtained free energy profiles are qualitatively in good agreement with the previous simulation^40^ as well as experimental results^46^. The deviations observed in the energetics are due to differences in force-fields and protocol followed (section 1.1 of SI). For the sake of comparison, we have also calculated the free energy profile of a water molecule adjacent to the mica surface. From the free energy profiles, we observed that Rb^+^ and K^+^ ions have single free energy minimum, whereas Na^+^ ion, both divalent ions (Mg^2+^ and Sr^2+^) and water molecules (Ow) have two minima in the free energy profiles within the AR. The location of these minima and corresponding adsorption strength (Δ*A* - the free energy difference between the minimum and bulk region) are listed in the Table 1. The cations and water molecules adsorb at these free energy minima locations as observed in the density profiles (Figs 1c,d and S1–S4 of SI).

From the free energy profiles, we found that K^+^ and Rb^+^ ions have similar adsorption strength, which suggests that their adsorption should be competitive in nature. Further, the adsorption strength of Na^+^ is weaker as compared to Rb^+^, and hence responsible for lesser adsorption of Na^+^ ions as compared to Rb^+^ ions. The free energy of water molecules adsorbed closer to the surface in the ditrigonal cavity (referred to as L1 water molecules, Fig. 1d) is much lesser as compared to all cations suggesting that adsorption of cations should occur at the expense of these L1 water molecules. As hypothesized, the adsorption strength of divalent Mg^2+^ ions is higher than the three monovalent ions. However, in case of Sr^2+^ ions, we have observed adsorption strength to be comparable to the monovalent ions. While free energy explains the difference in adsorption loading between monovalent Na^+^ vs Rb^+^ ions, however, it does not provide the reason for the lesser adsorption loading of divalent ions and different isotherm behavior observed. In order to understand that, we turn towards the detailed structural analysis of adsorbed ions using coverage, ion-hydration number, and cation-anion cluster formation. Also, to illustrate the role of hydration energies, we discuss the adsorption mechanism of Rb^+^, Na^+^, and divalent (Sr^2+^ and Mg^2+^) ions which have hydration energies similar, moderately higher and extremely higher compared to competitive surface K^+^ ions.

### Adsorption Behavior of Monovalent Ions

#### Mica–RbCl system

**Figure 4:**
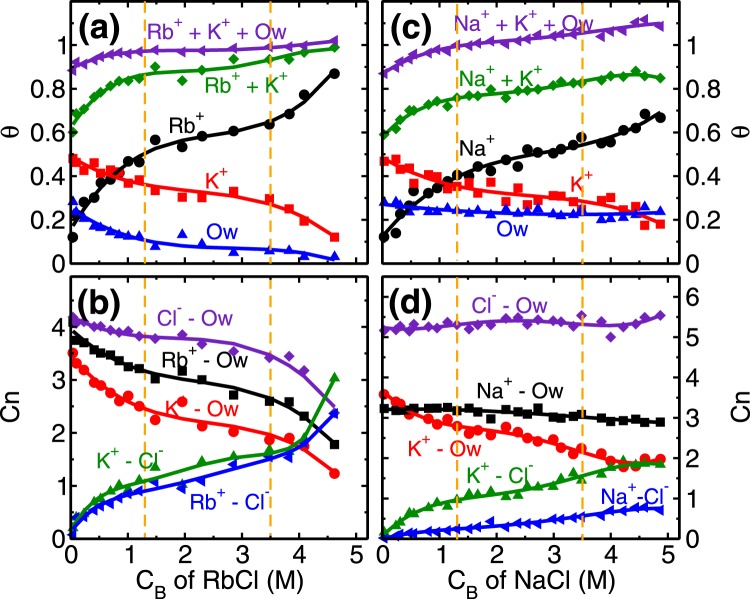
Coverage and co-ordination number of monovalent ions. **(a)** and **(c)** Variation in the surface coverage of water molecules and cations (*θ*_*i*_, *i* = *Ow* − *water*, *K*^+^ *and Rb*^+^/*Na*^+^ ion) with respect to the bulk concentration (C_*B*_) of RbCl and NaCl aqueous solution. At C_*B*_ = 0, ${\theta }_{K}\simeq {\theta }_{Ow}\simeq 0.5$. Ion coverage of *θ*_*Rb*/*Na*_ + *θ*_*K*_ = 0.5 corresponds to the total surface charge compensation whereas *θ*_*K*_ + *θ*_*Rb*/*Na*_ + *θ*_*Ow*_ = 1.0 corresponds to each adsorption site (ditrigonal cavity) occupied by the cations or water molecules. **(b)** and **(d)** Variation in the water co-ordination number (${C}_{n}^{i-Ow}$, *i* = *K*^+^, *Rb*^+^/*Na*^+^ and Cl^−^ ion) and anion co-ordination number (${C}_{n}^{i-Cl}$, *i* = *K*^+^ *and Rb*^+^/*Na*^+^ ion) with respect to C_*B*_ of RbCl and NaCl aqueous systems. ${C}_{n}^{Rb-Ow}$ decreases whereas ${C}_{n}^{Na-Ow}$ remains constant with increase in C_*B*_, illustrating the effect of hydration energy (Δ*G*_*hyd*,*Na*_ < Δ*G*_*hyd*,*Rb*_). The vertical dotted lines are used to indicate various adsorption isotherm regimes.

Every unit cell of mica surface contains two ditrigonal cavities (Fig. 1b) where cations and water molecules compete to adsorb, and hence taken as adsorption sites in the coverage analysis. In distilled water system (i.e. no salt present), K^+^ ions occupy half of the cavities (i.e. fractional coverage, *θ*_*K*_ ~ 0.5), and the rest are occupied by L1 water molecules (*θ*_*Ow*_ ~ 0.5). At the lowest RbCl salt concentration of (C_*Rb*,*B*_) 0.04 M studied here, Rb^+^ and K^+^ ions adsorb on the cavities and respectively occupy around 0.17 and 0.48 fraction of total cavities (Fig. 4a) while coverage of L1 water molecules is decreased to *θ*_*Ow*_ ~ 0.25. This adsorption of Rb^+^ and K^+^ ions is driven by their adsorption strength (Δ*A*) at the mica surface. Our calculations showed (Fig. 3b) an insignificant difference in their adsorption strength (Δ*A*_*Rb*_ ~ Δ*A*_*K*_), means both of them have an equal chance to occupy an adsorption site. As a result, we observed K^+^ ions were able to maintain their fractional coverage (of *θ*_*K*_ ~ 0.48) in the presence of Rb^+^ ions. Furthermore, lesser adsorption strength of L1 water molecules compared to both, Rb^+^ and K^+^ cations (Fig. 3a,b and Table 1) leads to their desorption from the surface. In this regime, the adsorption of Rb^+^ ions (*θ*_*Rb*_ = 0.5), occurs at a minimal desorption of K^+^ ions (${\rm{\Delta }}{\theta }_{K}=0.13$) and significant desorption of water molecules (${\rm{\Delta }}{\theta }_{Ow}=0.4$) highlighting the role of similar hydration energy of Rb^+^ and K^+^ ions (Table 1). The adsorption of Rb^+^ ions in this regime is governed by the attraction from the mica surface.

**Figure 5:**
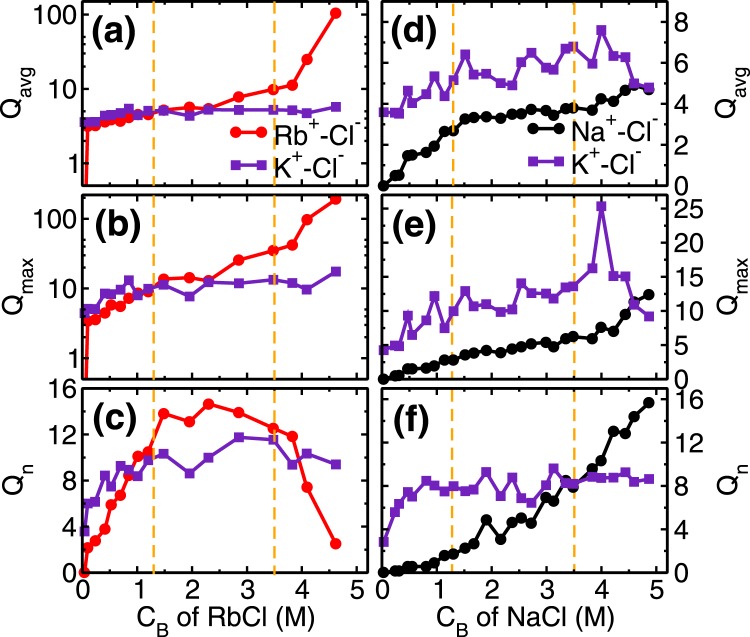
Cluster analysis of monovalent ions. **(a)** and **(d)** Average cluster size (Q_*avg*_), **(b)** and **(e)** maximum cluster size (Q_*max*_) and, **(c)** and **(f)** number of clusters (Q_*n*_) formed in the adsorption region at various C_*B*_ of RbCl and NaCl aqueous solution, respectively. The vertical dotted lines are used to indicated various adsorption isotherm regimes. The salting out behavior of RbCl leads to significant increase in their Q_*avg*_ and Q_*max*_ in third regime (C_*B*_ ≥ 3.5 M) due to conjoining of smaller clusters, which results in decrease in Q_*n*_ of RbCl clusters. The Q_*avg*_ of NaCl is limited to ~4 even at higher C_*B*_ indicating absence of salt formation.

The next adsorption regime (i.e. 1.3 ≤ C_*Rb*,*B*_ < 3.5 M) corresponds to the transition regime where predominantly the development of RbCl and KCl clusters occur. The total adsorption of (both, K^+^ and Rb^+^) cations beyond fractional coverage of (*θ*_*K*_ + *θ*_*Rb*_>) 0.5 creates an overcharge on the mica surface. This surface overcharging is compensated by the adsorption of Cl^−^ ions observed around 4 Å adjacent to the mica surface (Fig. S1 of SI). The association of these anions (Cl^−^) with adsorbed cations leads to a formation of salt clusters (i.e. RbCl and KCl) on the mica surface. We analyzed the average (Q_*avg*_), maximum (Q_*max*_) and the total number of clusters (Q_*n*_) formed by ion-pairs in the cluster calculations. Ion-pairs are defined as the combination of the cation-anion pair located within the first minimum of cation-anion pair correlation function (PCF). A collection of such ions forming a network of ion-pairs having a minimum three ions are defined as the cluster (i.e. excluding monomers and dimers). In the first adsorption regime, while the Cl^−^ ions do associate with Rb^+^ ions and form around ${Q}_{n}\simeq 10$ clusters (Fig. 5c), nevertheless *Q*_*avg*_ of RbCl and KCl clusters remain around 5 (Fig. 5a). In the second regime, these small clusters are conjoined by the significant adsorption of Cl^−^ ions, leading to the formation of bigger clusters. During this regime, we observed that *Q*_*avg*_ and *Q*_*max*_ of RbCl grows from around 5 to 10 and 9 to 35, respectively (Fig. 5a,b). Whereas, *Q*_*avg*_ and *Q*_*max*_ of KCl remained constant at around 5 and 12 respectively. The association of Cl^−^ with adsorbed cations is further confirmed by the coordination number analysis (Fig. 4b), where coordination number is defined as the number of anions (or water molecules) present within the first minimum of PCF. We observed an increase in the ion-Cl^−^ coordination number (${C}_{n}^{ion-Cl}$) and decrease in the ion-water coordination number (${C}_{n}^{ion-Ow}$) of both Rb^+^ and K^+^ cations. Since both of these ions have similar hydration energy hence the rate of change of coordination number (i.e. decrease in ${C}_{n}^{ion-Ow}$ and increase in ${C}_{n}^{ion-Cl}$) is similar in both of the ions. This suggests that cation hydration energy plays a crucial role. During this regime as well, the adsorption of Rb^+^ ions (*θ*_*Rb*_ = 0.46−0.64) occurs by the desorption of energetically less favorable L1 water molecules $({\theta }_{Ow}=0.12-\mathrm{0.06)}$, while the K^+^ ions are retained $({\theta }_{K}\simeq \mathrm{0.3)}$ (Fig. 4a).

**Figure 6:**
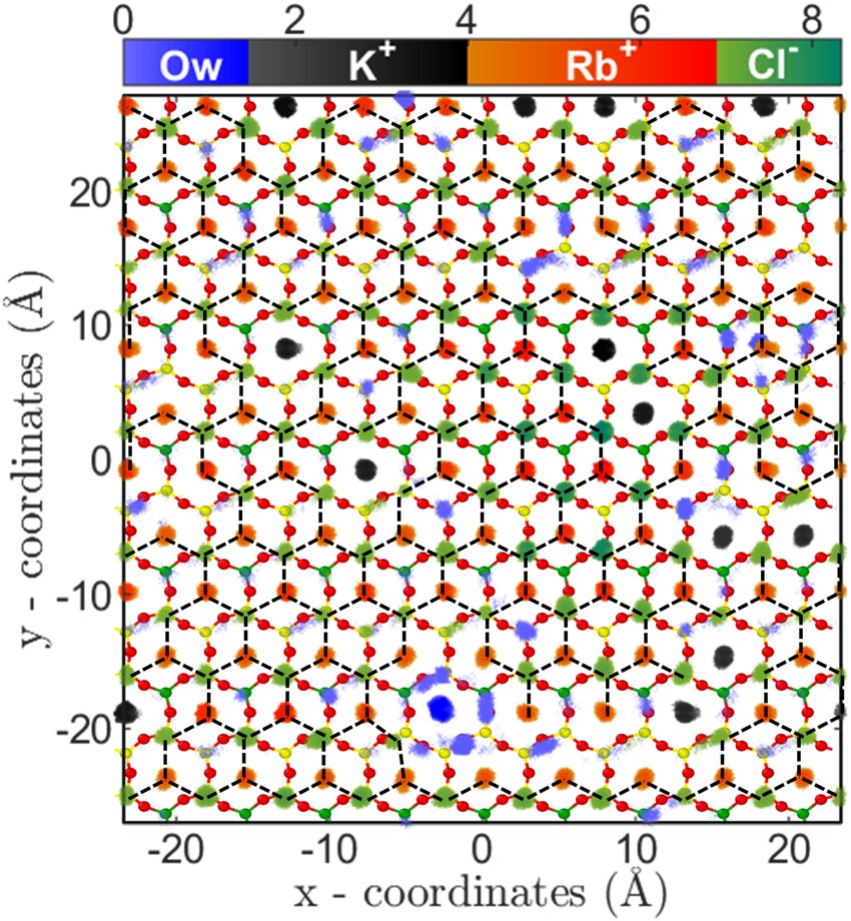
Arrangement of water molecules and ions, parallel to the surface in RbCl system. Two-dimensional density distribution of water, K^+^, Rb^+^ and Cl^−^ ions present within AR (in Å^−3^) at C_*Rb*,*B*_ = 4.62 M are shown using separate colorbars. Background is the top view of mica surface consisting of silicon(yellow), aluminum(green) and basal oxygen atoms(red). K^+^ and Rb^+^ ions are adsorbed on the ditrigonal cavities, whereas Cl^−^ ions adsorb on top of silicon (Si) atom. Each Rb^+^ and K^+^ ion is surrounded by three Cl^−^ ions forming RbCl and KCl cluster. Surface mediated salting out of RbCl occurs leading to formation of a giant RbCl cluster of size ~105 spanning the entire surface (shown by dotted lines).

The third regime of RbCl adsorption isotherm (i.e. C_*Rb*,*B*_ ≥ 3.5 M) is mainly due to (a) salting out of RbCl near the mica surface and (b) increased concentration (and hence chemical potential) of Rb^+^ ions in bulk solution. The salting out behavior is confirmed by the cluster size analysis (Fig. 5a–c), where, the *Q*_*avg*_ and ${Q}_{max}$ of RbCl cluster increased to around 105 and 190 from 10 and 35, respectively(Fig. 5a,b). Due to the formation of the bigger cluster, the *Q*_*n*_ decreased from ~12.5 to 2.5 (Fig. 5c). The two-dimensional (2D) density distribution of ions and water present in the AR at C_*Rb*,*B*_ = 4.62 M shows that RbCl salt are arranged in triangular type lattice on the mica surface (Fig. 6). The Rb^+^ ions adsorbed on the cavities are surrounded by 3 Cl^−^ ions adsorbed on top of the silicon (Si) atom of mica surface. This is the first computational study which shows surface mediated early salting of RbCl at 4.62 M compared to the salt formation in bulk solution at solubility limit of 7.52 M. Since the number of adsorbed L1 water molecules are minimal, hence the adsorption of Rb^+^ ions in this regime (${\theta }_{Rb}=0.64-0.87$) occurs by the desorption of K^+^ ions (*θ*_*K*_ = 0.3−0.12). The *Q*_*n*_, *Q*_*avg*_ and *Q*_*max*_ of KCl clusters remains constant during this third regime (Fig. 5a–c). In the cluster calculations, the monomer and dimers are not included and the invariant cluster size parameters (*Q*_*avg*_, *Q*_*max*_ and *Q*_*n*_) of KCl cluster at higher concentration indicate that the monomers and dimers of K^+^-Cl^−^ ion-pairs are desorbing in this regime.

In summary, Rb^+^ ions adsorption on the mica surface in the three regimes is respectively governed by (a) attraction from the mica surface, (b) development of RbCl cluster and (c) salting out behavior at a higher C_*B*_. Due to similar hydration energy of Rb^+^ ions with competing K^+^ ions, the desorption of K^+^ ions is slow as compared to less favorable L1 water molecules. The mechanism of Rb^+^ ions adsorption occurs mainly by the desorption of adsorbed water molecules, and at higher concentration by the desorption of K^+^ ions from the surface.

#### Mica–NaCl system

The adsorption behavior of Na^+^ ion on mica surface is similar to Rb^+^ ions, as explained above. Here also we observed three adsorption regime (Fig. 2), with adsorption of Na^+^ ions in the first regime governed by the attraction from the surface, and development of NaCl cluster in the later stages (Fig. 5d–f). The main difference is the higher hydration energy of Na^+^ ion compared to Rb^+^ and K^+^ ion (Table 1). Due to this significant difference, we hypothesize that interfacial water should play a crucial role in the adsorption of Na^+^ ions here. Indeed we find five noteworthy observations related to interfacial water in Na^+^ ions adsorption which are absent in Rb^+^ ion case. (a) Retention of L1 water molecules (${\theta }_{Ow}$) even at higher C_*Na*,*B*_, (b) total fractional coverage (i.e. *θ*_*Na*_ + *θ*_*K*_ + *θ*_*Ow*_) exceeding 1, (c) higher number of water molecules in the AR in Na^+^ system compared to Rb^+^ system, (d) constant ${C}_{n}^{Na-Ow}$ at all C_*Na*,*B*_ and (e) absence of significant NaCl cluster. The reasons for these observations are explained below.

In our analysis, we observed that ${\theta }_{Ow}\sim 0.23$ (L1 water molecules) at all NaCl concentrations studied (Fig. 4c). These L1 water molecules are found in two configurations; either solely adsorbed on the cavities or co-adsorbed with Na^+^ ions as part of cations hydration shell (Fig. S12 of SI). This co-adsorption of water is possible due to smaller hydration shell size (Fig. S6 of SI) and higher hydration energies of Na^+^ ions (Table 1) compared to K^+^ and Rb^+^ ions. With the increase in concentration, the fraction of water molecules co-adsorbed with Na^+^ ions increases linearly (Table S2 of SI). As a result, the total fractional coverage of adsorbed species (i.e. *θ*_*Na*_ + *θ*_*K*_ + *θ*_*Ow*_) exceeds 1.0 at higher concentrations (Fig. 4c).

To understand the constant profile of ${C}_{n}^{Na-Ow}$, we probed the total number of water molecules in AR. We found that presence of a higher number of water molecules and their redistribution from K^+^ to Na^+^ hydration shell is responsible for this behavior (Figs S9 and S10 of SI) which is explained below. Our previous work has shown that, while the majority of the L2 and L3 water molecules (Fig. 1d) hydrate the adsorbed cations (K^+^ and Na^+^/Rb^+^), however a small fraction of free (i.e. non-hydrating) water molecules are also present. The presence of these free water molecules is essential to form a network of hydrogen bonds with all molecules of L2 + L3 layer and especially with L1 water molecules, thus stabilizing L2 and L3 layer^35,38^. Since the number of L1 water molecules is significant in Na^+^ ions, this leads to the higher fraction of free water molecules of L2 and L3 layer even at higher salt concentration of Na^+^ ions as compared to the case of Rb^+^ ions (Fig. S9 of SI). In addition, though the number of K^+^ ions and water molecules hydrating them decreases with an increase in *C*_*Na*,*B*_, however, the total number of hydrated water molecules (i.e. hydrating Na^+^ and K^+^ ions) is found to remain constant at all ${C}_{Na,B}$ (Table S2 in SI). This suggests that water molecules are re-distributing from K^+^ hydration to Na^+^ hydration shell, motivated by their hydration energies (Fig. S10 of SI).

Since Na^+^ ions are preferentially hydrated by water molecules, Cl^−^ ions adsorb more near the K^+^ ions and preferably form KCl cluster as compared to NaCl cluster. This is confirmed in the cluster size analysis where we observe that *Q*_*avg*_, *Q*_*max*_ and *Q*_*n*_ of NaCl cluster is less than KCl cluster (Fig. 5d–f). The adsorption of Na^+^ ion in the third regime of adsorption isotherm is governed by (a) increased ${C}_{Na,B}$, which forces Na^+^ ions to adsorb on the surface, and (b) subsequent desorption of K^+^ ions. Both of these phenomena is captured in cluster size analysis, where a decrease in KCl cluster size (especially *Q*_*max*_), and corresponding increase in *Q*_*avg*_, *Q*_*max*_ and ${Q}_{n}$ of NaCl cluster is observed(Fig. 5d–f). In the RbCl system, the Rb^+^ adsorption in the third regime occurs due to desorption of both water molecules and K^+^ ions, whereas here in NaCl system K^+^ ions desorption is only the limiting factor. Due to higher hydration energy of Na^+^ ions, the water molecules are retained in the hydration shell whereas desorption of K^+^ ions occur.

### Adsorption Behavior of Divalent Ions

**Figure 7:**
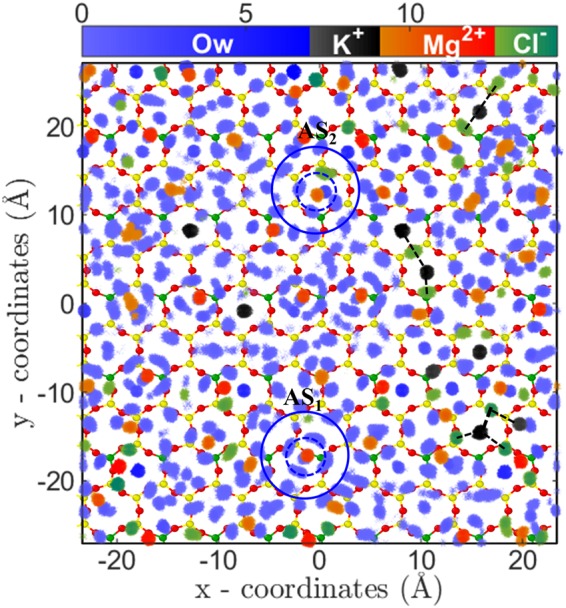
Arrangement of water molecules and ions, parallel to the surface in MgCl_2_ system. Two-dimensional density distribution of water, K^+^, Mg^2+^ and Cl^−^ ions present within AR (in Å^−3^) at C_*Mg*,*B*_ = 3.80 M. Legends are same as Fig. 6. Mg^2+^ ions adsorb near bridging oxygen atoms (AS_1_) as well as on the ditrigonal cavities (AS_2_). Water molecules form a tetragonal hydration shell around divalent ions (first and second hydration shell are shown by dotted and continuous circle). Ion-hydration water adsorb in nearby cavities and restricts adsorption of other cations via steric repulsion. This leads to lesser adsorption of divalent ions and early saturation of adsorption isotherm. Similar behavior is observed for Sr^2+^ divalent ion as well (Fig. S12 in SI).

Since divalent ions have higher electrostatic interactions compared to monovalent ions, we expected their adsorption quantity would be higher as compared to monovalent ions. However, we found two remarkable counter-intuitive observations from the adsorption isotherms of divalent ions; (a) low adsorption quantity and (b) early saturation behavior as compared to monovalent ions (Fig. 2). In addition, we observed that adsorption isotherm of both Mg^2+^ and Sr^2+^ ions are similar. Since the divalent ions have higher hydration energy compared to monovalent ions (Table 1), it pointed that the observed behavior could be related to the hydration structure of adsorbed ions. To confirm this point, we investigated the ion-water PCF and 2D density distribution of adsorbed divalent ions near the mica surface. The ion-water PCF clearly shows a strong peak followed by the PCF going to zero indicating that water molecules of hydration shell are tightly bound to cation (Figs S7 and S8 of SI). The bulk analysis of these ions indicates that hydration water has higher residence time adjacent to these ions^47^. This indicates that divalent ions essentially have an effective higher size due to their tightly bound first hydration shell, which reduces the available area for the adsorption of ions. The 2D density distribution analysis also confirms the hypothesis where distinct water density peaks around ions are observed (Fig. 7). Further, the 2D density distribution shows that these ions are adsorbed on the cavities in two different states; near the surface oxygen (AS_1_) and at the center of cavity (AS_2_) (Figs 7, S13 and S14 of SI). The hydrated water molecules are found to adsorb in the nearby hexagonal cavities, which due to steric repulsion prevents adsorption of other cations (i.e., Mg^2+^/Sr^2+^ and K^+^). Due to this arrangement of divalent ions and their hydration water, the total number of hexagonal sites available for adsorption is reduced and hence leads to lesser adsorption and early saturation, as observed. Since both of these ions have comparable hydration energy and hydration structure, hence their adsorption isotherm (i.e. quantity) is also similar at various C_*B*_.

**Figure 8:**
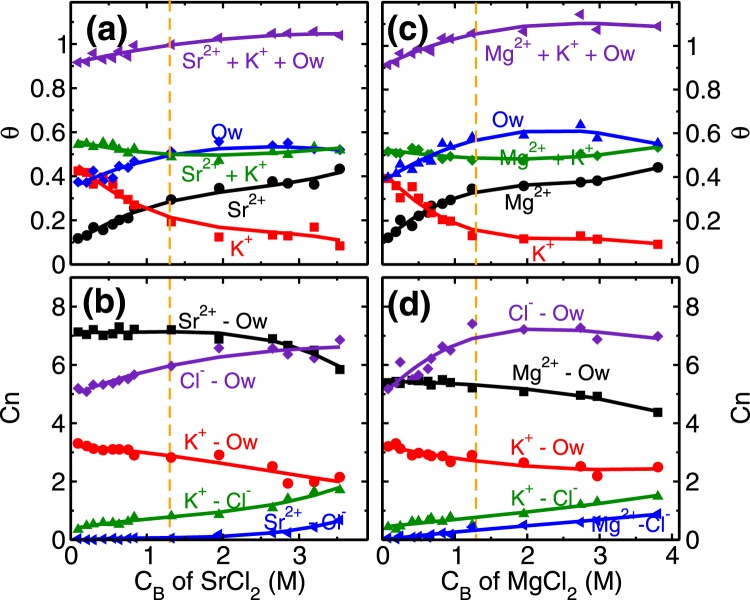
Coverage and co-ordination number of divalent ions. **(a)** and **(c)** Variation in the surface coverage of L1 water molecules and cations (*θ*_*i*_, *i* = *Ow* − *water*, *K*^+^ *and Sr*^2+^/*Mg*^2+^) with respect to the C_*B*_ of SrCl_2_ and MgCl_2_ aqueous solution. The increase in *θ*_*Ow*_ with increase in C_*B*_, signifies the effect of hydration energy of divalent cations. Adsorption of divalent ions, in both cases, occur primarily by desorption of surface K^+^ ions. **(b)** and **(d)** Variation in the water co-ordination number (${C}_{n}^{i-Ow},\,i\,=$ K^+^, Sr^2+^/Mg^2+^ and Cl^−^) and anion co-ordination number (${C}_{n}^{i-Cl},\,i\,=$ K^+^ and Sr^2+^/Mg^2+^) with respect to C_*B*_ of SrCl_2_ and MgCl_2_ aqueous solution.

Similar to the monovalent ions, here also we have performed coverage ($\theta$) and coordination number (C_*n*_) analysis of divalent ions at various C_*B*_. The role of higher hydration energy of divalent ions is very clear in this analysis. In the first regime of adsorption isotherm, (i.e. C_*B*_ < 1.3 M) the coverage of both divalent ions increases (Fig. 2). We find that the coverage of L1 water molecules also increases in divalent ions case (Fig. 8a,c) as opposed to decreasing and a constant trend observed in RbCl and NaCl systems (Fig. 4a,c). In the first regime itself, the total coverage of cations and L1 water molecules (i.e. *θ*_*M*_ + *θ*_*K*_ + *θ*_*Ow*_; M = Sr/Mg) reaches 1, with ${\theta }_{Ow}$ ~ 0.5 (Fig. 8a,c). From coverage analysis, it is clear that the adsorption of divalent ions in the first regime is governed by the surface attraction hence leads to the rapid rise in coverage. Whereas in the second regime (C_*B*_ ≥ 1.3 M), the adsorption occurs by the depletion of K^+^ ions. The cluster size analysis also confirms this hypothesis, where we find an insignificant number of M-Cl (where M = Sr/Mg) clusters formation in the first adsorption regime and a minimal number of clusters in the second regime (Fig. S15 in SI). This indicates that salting out behavior as observed in the RbCl system is not present in the divalent ions case. Again, the ion hydration energy plays a crucial role here. The ions prefer to be surrounded by water molecules (Fig. 7, Figs S13 and S14 of SI) as compared to Cl^−^ ions, hence the increase in coordination number (${C}_{n}^{M-Cl}$, *M* = Sr/Mg) is minimal in divalent ions case (Fig. 8b,d).

## Summary and Conclusion

We have performed series of MD simulations of monovalent (Rb^+^ and Na^+^) and divalent (Sr^2+^ and Mg^2+^) cations based salt solutions (*C*_*B*_ = 0–4.5 M) to investigate the role of cation charge, hydration energy, counter-ion (K^+^) competitiveness and co-ion (Cl^−^) association on their adsorption and structural behavior adjacent to mineral (mica) surface. We found that adsorption of monovalent ions occurs in 3 regimes; initial rapid increase for *C*_*B*_ < 1.3 M, followed by a slow increase between 1.3 ≤ *C*_*B*_ < 3.5 M and a rapid increase beyond *C*_*B*_ ≥ 3.5 M. Whereas divalent ions exhibit only 2 regime; initial rapid increase followed by early saturation. Despite having higher electrostatic attraction, adsorption of divalent ions is lesser as compared to monovalent ions.

The free energy calculation revealed that rapid rise in ion concentration near surface in the first regime of adsorption isotherms was due to surface attraction. Coverage and cluster analysis showed the association with Cl^−^ co-ions was the reason for a slow increase in the second regime as well as a rapid increase in the third regime due to cluster growth. In case of adsorbing cation (Rb^+^) which have lower hydration energy (and comparable to hydration energy of competitive surface K^+^ ion), they repel water molecules from AR and prefer to form large cation-anion (RbCl) clusters leading to surface mediated salting out behavior. For the cations (Na^+^) which have moderate hydration energy, they retain water molecules in their hydration shell and does not prefer to form cation-anion clusters. The divalent cations have very high hydration energy, due to which they form tight bound water hydration shell around them. This effectively increases their size which leads to lesser adsorption and early saturation.

The findings of this work, i.e. molecular understanding of adsorption of ions at the mineral-water interface, mechanism and role of hydration energy, have profound applications in toxic element contaminants in aquifers and surface water, plant nutrient supply and effectiveness of water purification method, where maximum ions adsorption on adsorbent is desirable. The obtained understanding would help in the better design of these adsorbate materials. The findings also have implications in super-capacitors, where ion adsorption on charged surfaces from electrolyte solution dictates their energy storage capacity. Our analysis finds that higher concentration of elecrolyte is not always desirable, rather hydration structure plays the crucial role.

### Methods: Molecular dynamics (MD) simulations

In muscovite mica (KAl_2_(Si_3_Al)O_10_(OH)_2_), the octahedral sheet(O) of aluminum(Al) is sandwiched between two silicon(Si) tetrahedral sheets(T) making mica as a TOT layered structure. An isomorphic substitution of one out of four Si^4+^ atom in the tetrahedral sheet by an Al ^3+^ atom yields to a permanent surface charge deficit of −1e per unit cell. This deficit charge is balanced by the cation, mostly potassium (K^+^) ion, which holds two TOT layer together by electrostatic attraction. A cleaved mica has an atomically smooth surface with the exposed hexagonal ring structure formed by sharing of basal oxygen atoms between Si and Al atoms. In this work 9 × 6 × 2 unit cell of muscovite mica was created using the X-ray data^48^, which gives a mica slab of $46.86\times 54.1\times 20.0$ Å.

The simulation system was constructed such that the surface of muscovite mica (001 plane) was in direct contact with aqueous solution. Interactions between muscovite mica atoms were described using the CLAYFF force field^49^. Water molecules in the systems were represented using simple point charge extension (SPC/E) model^50^. In this work potential parameters for Na^+^ and Cl^−^ ions were taken from Smith and Dang^51^, Rb^+^ ion from Joung and Cheatham^52^, Mg^2+^ ion from Aqvist^53^ and Sr^2+^ ion from Mamatkulov *et al*.^54^ (Table S3 in SI). The initial configuration of the aqueous systems were created using the Packmol software^55^. Open source LAMMPS package was used to perform MD simulations in an NVT ensemble^56^. Periodic boundary conditions were applied in the lateral *x* and *y* directions. The system is non-periodic in *z* - direction and has a length of 300 Å, out of which around 20 Å is the mica surface, 65 Å is the liquid region and rest is the vapor region. The simulation box was bounded by a repulsive graphene sheet at the vapor side. The temperature of the system was maintained at 298 K using Nose-Hoover thermostat^57,58^. The long-range Coulombic interactions were calculated using the particle-particle particle-mesh method for the slab system having vacuum size of 636 Å and accuracy of 10^−4^. The Lennard-Jones interactions and short-range part of Coulombic interactions were calculated with a cutoff distance of 12.5 Å. The rigidity of the water molecule was maintained using SHAKE algorithm^59^. All mica atoms except surface K^+^ ions were kept fixed in all simulations. The systems were equilibrated for 30 ns followed by a production run of 5 ns with a step size of 2 fs. The finite size effect was investigated by studying few larger NaCl systems and the results were found to be invariant as shown in Fig. 2.

## Electronic supplementary material

Supplementary Information
