# Supplementary material for: Role of hydration energy and co-ions association on monovalent and divalent cations adsorption at mica-aqueous interface
Source: Sci Rep. 2018 Aug 15;8:12198. doi: 10.1038/s41598-018-30549-9 (PMC6093886; doi:10.1038/s41598-018-30549-9)
Supplement: Supplementary file 1 — Supplementary Information [file 41598_2018_30549_MOESM1_ESM.pdf]

Supplementary Information: Role of hydration energy and co-ions association on monovalent and divalent cations adsorption at mica-aqueous interface

Sai Adapa and Ateeque Malani*

Department of Chemical Engineering, Indian Institute of Technology Bombay, Mumbai, MH 400076, India

Contents

S1. Free energy calculation **2**
S1.1. Simulation details **2**
S2. Atomic densities **3**
S3. Pair correlation function (PCF) **7**
S4. Two-dimensional (2D) density distribution near mica surface **16**
S5. Cluster size analysis of divalent ions **20**
References **21**

List of Figures

S1 Density profiles of ions and water in mica-RbCl systems . . . 3
S2 Density profiles of ions and water in mica-NaCl systems . . . 4
S3 Density profiles of ions and water in mica-$SrCl_2$ systems . . . 5
S4 Density profiles of ions and water in mica-$MgCl_2$ systems . . . 6
S5 Pair correlation functions and coordination number in mica-RbCl systems . . . 8
S6 Pair correlation functions and coordination number in mica-NaCl systems . . . 9
S7 Pair correlation functions and coordination number in mica-$SrCl_2$ systems . . . 10
S8 Pair correlation functions and coordination number in mica-$MgCl_2$ systems . . . 11
S9 Number of water molecules in the adsorption region of mica-RbCl systems . . . 14
S10 Number of water molecules in the adsorption region of mica-NaCl systems . . . 15
S11 Two-dimensional density distribution of ions and water in mica-RbCl systems . . . 16
S12 Two-dimensional density distribution of ions and water in mica-NaCl systems . . . 17
S13 Two-dimensional density distribution of ions and water in mica-$SrCl_2$ systems . . . 18
S14 Two-dimensional density distribution of ions and water in mica-$MgCl_2$ systems . . . 19
S15 Cluster analysis of mica-$SrCl_2$ systems . . . 20

List of Tables

S1 Hydration and free water molecules present in the adsorption region of mica-RbCl systems . . . 12
S2 Hydration and free water molecules present in the adsorption region of mica-NaCl systems . . . 13
S3 Potential parameters . . . 21

*Corresponding author: Email: amalani@iitb.ac.in, Phone:+91-22-2576-7205

S1. Free energy calculation

The Free energy profile of water molecule and an ion adjacent to the mica surface was calculated using umbrella sampling simulations.[1,2] The movement of the atom was restricted in the z-direction using harmonic potential,

$$U^b(z) = \frac{k_f}{2}(z - z_o)^2, \tag{1}$$

where k_f is the force constant and z_o is the center of sampling window. Weighted histogram analysis method (WHAM) was used to obtain free energy profiles from individual sampling windows[3]. In WHAM, the free energy profiles are generated by solving iteratively self consistent equations;

$$e^{-\beta F_i} = \int e^{-\beta U_i^b(z)} \langle \rho^u(z) \rangle \, dz \tag{2}$$

$$\langle \rho^u(z) \rangle = \frac{\sum_{i=1}^{N_W} n_i \langle \rho^b(z) \rangle_i}{\sum_{j=1}^{N_W} n_j e^{-\beta\left[U_j^b(z) - F_j\right]}} \quad . \tag{3}$$

Where i is the sampling window, $\rho^u(z)$ is the unbiased probability distribution, $\rho^b(z)$ is the biased probability distribution computed using n_i independent data points and F_j is the free energy in the jth window. This iterative procedure was repeated until relative free energy (F_j) of 10^{-9} in each window was achieved.

S1.1. Simulation details

We performed free energy calculation in a simulation cell consisting of mica surface, respective surface ion and water molecule. In these simulations no extra salt is added. We randomly pick a cation/water molecule and restrained them at various positions using umbrella potential. The data was collected with an interval size of 0.1 Å for distance $0-16$ Å between cation/water and mica surface by performing bias umbrella sampling with force constant of $k_f = 250$ kcal mol^{-1} Å^{-2}. At each interval, we carried out 500 ps equilibration steps followed by 100 ps production steps using timestep of 1 fs. The biased density distributions obtained from umbrella sampling are stitched together using WHAM to obtain free energy profile. The simulations were performed using open source LAMMPS software with colvars script.[4]

We have also performed sensitivity analysis of force constant k_f on free energy profile and found that for $k_f \geq 250$ kcal mol^{-1} Å^{-2} provides consistent results, hence chosen for all remaining simulations. During our simulation, unlike Kobayashi et al.[5], we let the cation to move freely in the lateral (x and y) directions to access all possible hydration and adsorption states. This leads to a small variation in our observed values of free energy compared to their data.

S2. Atomic densities

The structural details at the mica-water interface was obtained by calculating the atomic density distribution as,

$$\rho_i(z) = \left\langle \sum_{j=1}^{N_i} \delta(z - z_j) \right\rangle , \tag{4}$$

where N_i is the number of i atoms in the system and angular bracket $\langle \cdots \rangle$ indicates time average. The delta function is numerically evaluated as $\delta(z-z_j) = \dfrac{\theta(z-\Delta z/2 - z_j) - \theta(z+\Delta z/2 - z_j)}{A\ \Delta z}$, where $\theta(z)$ is a Heaviside step-function, Δz is the bin thickness in z-direction and A is the area in the lateral directions ($L_x \times L_y$). The average concentration in the specific region was evaluated as,

$$C_i = \frac{1}{z_u - z_l} \int_{z_l}^{z_u} \rho_i(z)\ dz, \tag{5}$$

where z_l and z_u are the boundaries of the interested region decided based on water density profile.

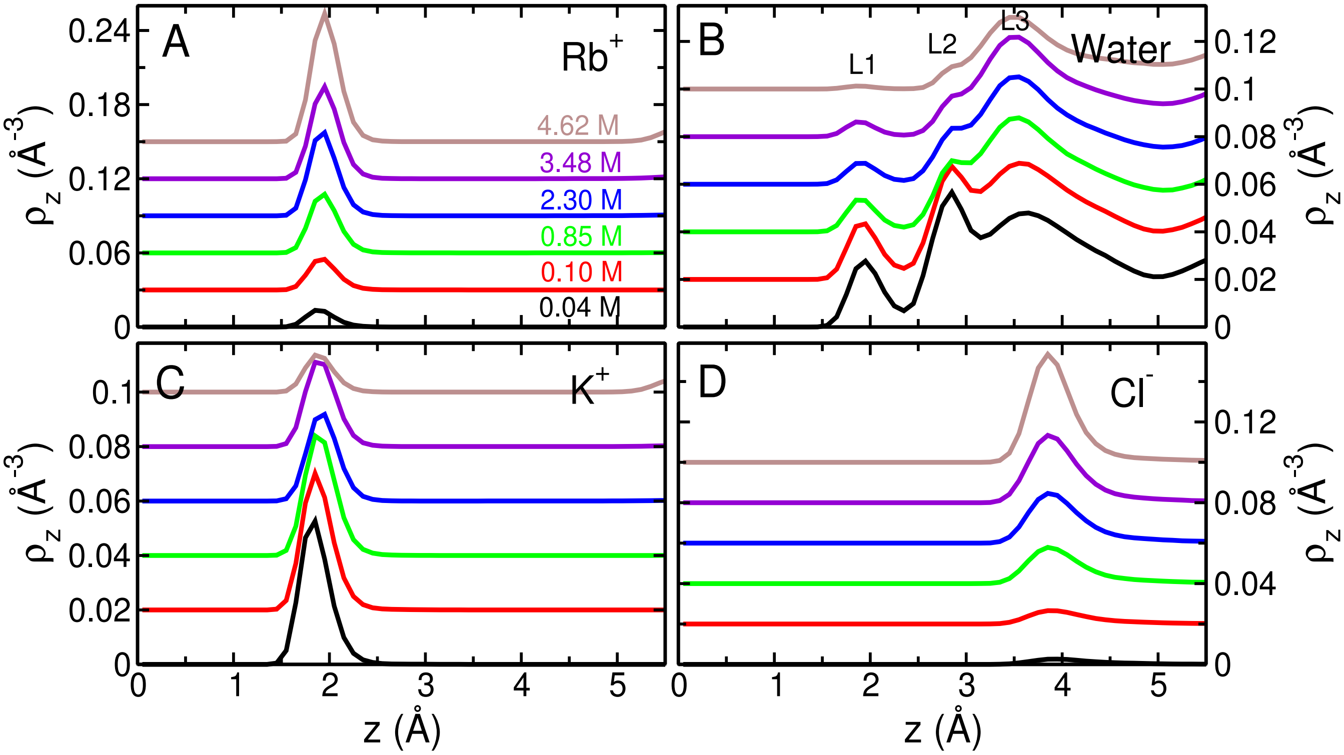

Figure S1. Density profile of (A) Rb^+ ions, (B) water (Oxygen atom), (C) K^+ ions and (D) Cl^- ions adjacent to the mica surface at different bulk RbCl salt concentration, $C_{Rb,B}$. The adsorption region (AR) is defined as 0−5 Å adjacent to mica surface. For clarity, density profiles are shifted along y−axis. The reference, i.e. $z = 0$, corresponds to the plane passing through basal bridging oxygens of mica surface. Both, Rb^+ and K^+ ions are adsorbed as single density peak at around 1.85 Å from the mica surface, whereas Cl^- ions are adsorbed at around 4 Å. The adsorption location of cations and water molecule is consistent with the location of minima observed in the free energy profiles. The water molecules present in the first peak (around 1.85 Å) are adsorbed on the ditrigonal cavities (referred to as L1 water molecules). The remaining water molecules (L2 + L3 layer) present within the AR are mainly due to either hydrating the adsorbed ions or forming hydrogen bond among themselves. In B, desorption of the water from ditrigonal cavities (L1 water molecules) is responsible for the decrease in L2 layer water molecules. At highest $C_{Rb,B}$ few water molecules are present in the L3 layer due to salting out of RbCl on the mica surface.

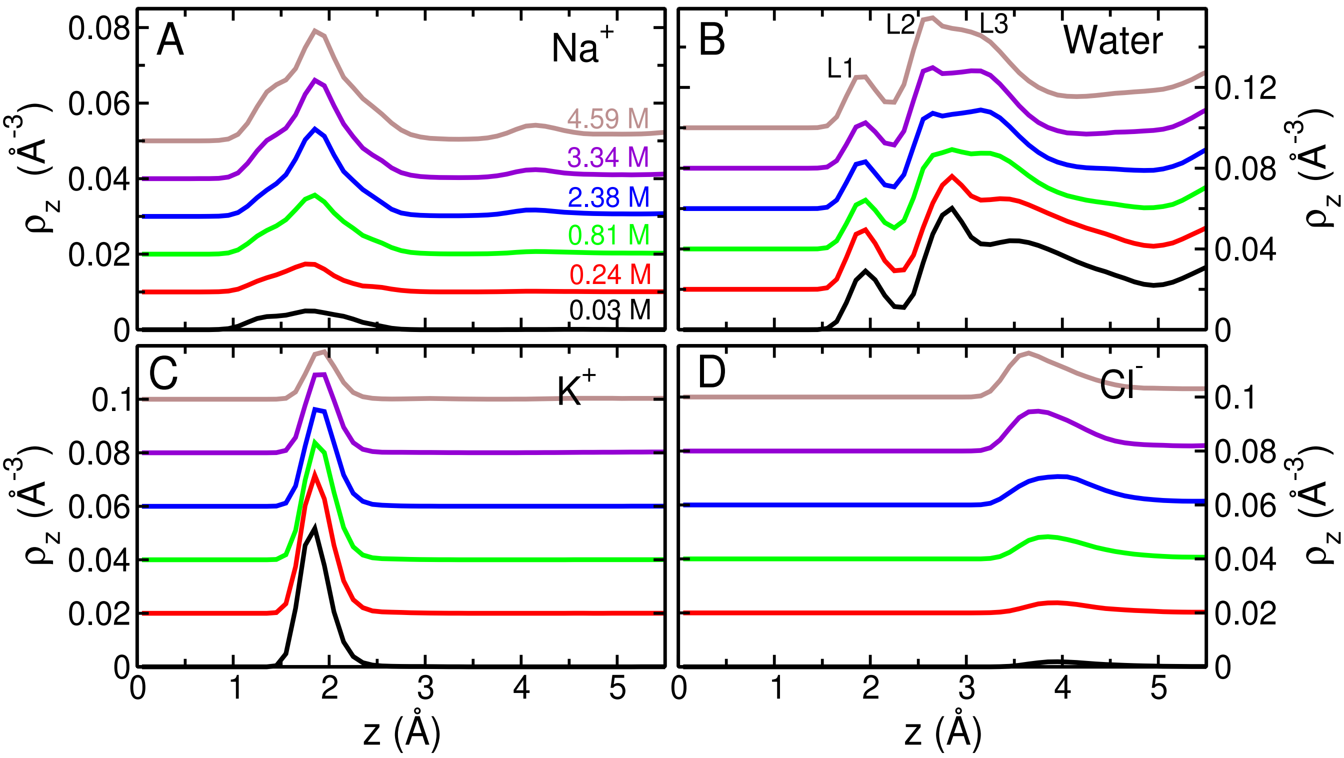

Figure S2. Density profile of (*A*) Na^+ ions, (*B*) water (Oxygen atom), (*C*) K^+ ions and (*D*) Cl^- ions adjacent to the mica surface at different bulk NaCl salt concentration, $C_{Na,B}$. The adsorbed Na^+ ions are distributed between 1 - 3 Å and at higher concentration ($C_{Na,B} \geq 2.38$ M), ions adsorb at 4.2 Å as well. The K^+ ions are adsorbed at 1.80 Å and the decrease in peak height at higher $C_{Na,B}$ is due to the desorption of K^+ ions from the mica surface. The Cl^- ions are adsorbed at around 4 Å from the mica surface. These adsorption position of cations are in good agreement with the location of minima observed in the free energy profiles of the respective ions. The merging of L2 and L3 water density peak (in subfigure *B*) is due to smaller hydration shell size of Na^+ ions compared to K^+ ions.

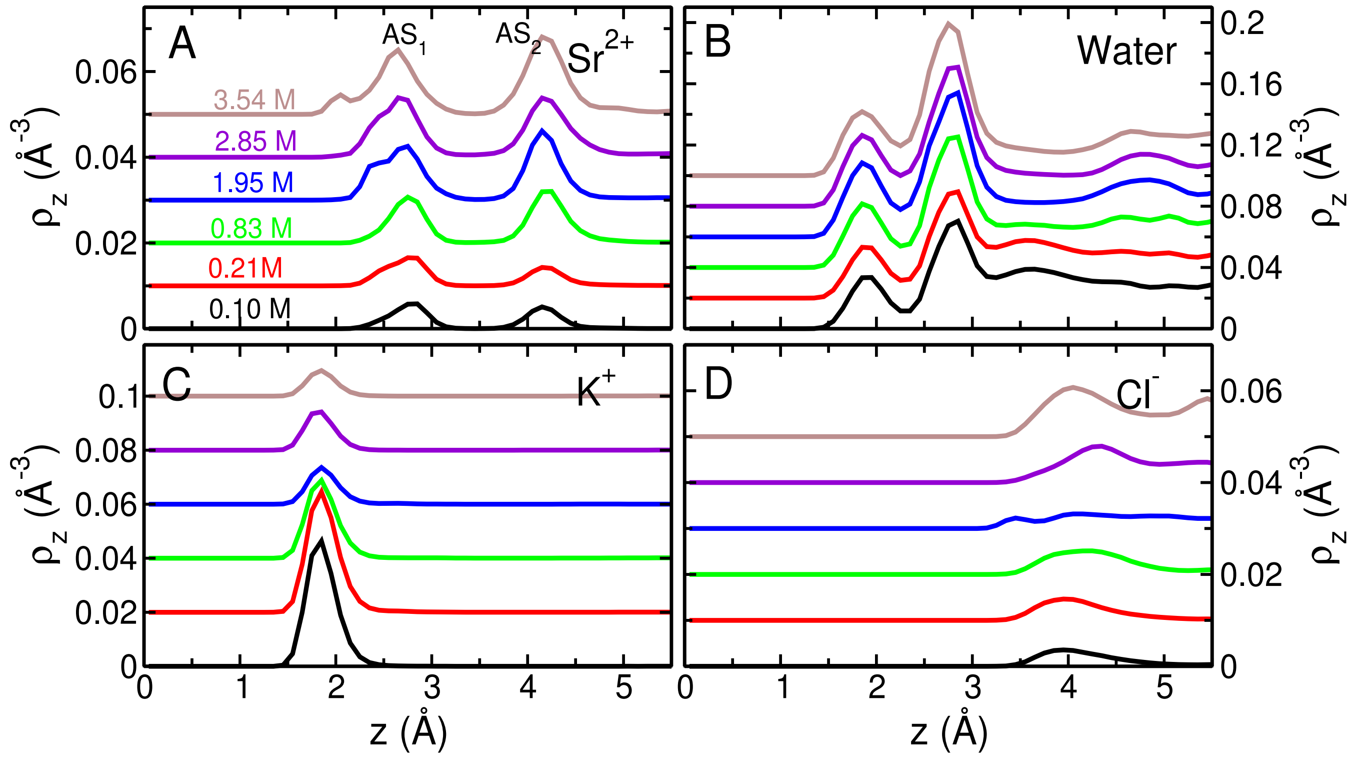

Figure S3. Density profile of (*A*) Sr^{2+} ions, (*B*) water (oxygen atom), (*C*) K^{+} ions and (*D*) Cl^{-} ions normal to the mica surface at different $C_{Sr,B}$ values. The Sr^{2+}ions adsorb at two different locations within the AR (referred to as AS_1 and AS_2). Adsorption of Sr^{2+} ions occurs simultaneously in both AS_1 and AS_2 due to lesser energy barrier ($\sim$ 5.6 kcal mol^{-1}) between these two minima position.

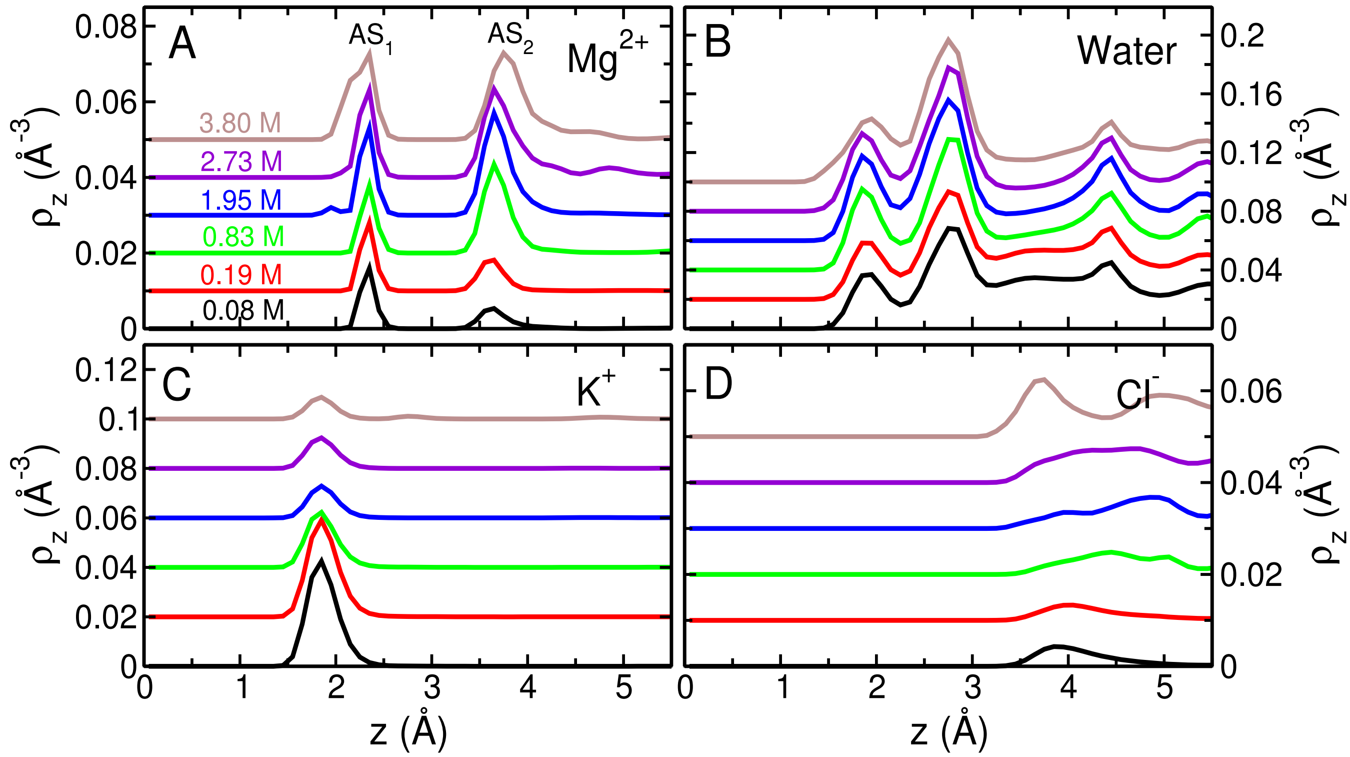

Figure S4. Density profile of (*A*) Mg^{2+} ions, (*B*) water (oxygen atom), (*C*) K^{+} ions and (*D*) Cl^{-} ions normal to the mica surface at different $C_{Mg,B}$ values. Similar to Sr^{2+} ions, here also Mg^{2+} ions are adsorbed in AS_1 and AS_2 states. Adsorption of Mg^{2+} ions in AS_1 is preferred over AS_2 at lower concentrations due stronger adsorption strength of AS_1 and higher energy barrier between two states ($\sim$ 10 kcal mol^{-1}).

S3. Pair correlation function (PCF)

The pair correlation function between atoms i and j was calculated as,

$$g_{ij}(r) = \frac{1}{\rho_j} \left\langle \frac{1}{N_i} \sum_{i=1}^{N_i} \sum_{\substack{j=1 \\ i \neq j}}^{N_j} \delta\left(r - r_{ij}\right) \right\rangle , \tag{6}$$

where, r_{ij} is the distance between atom i and j and ρ_j is the density of j type atom in system. The delta function was evaluated numerically as $\delta(r - r_{ij}) = \dfrac{\theta\left(r - \Delta r/2 - r_{ij}\right) - \theta\left(r + \Delta r/2 - r_{ij}\right)}{4\pi r^2 \Delta r}$, where, Δr is the bin thickness of spherical shell. When PCF between cation (i-type) and anion/water molecule (j-type) is evaluated within specific region (AR or bulk region), we ensured that cation is present in that region whereas no strict restriction is applied on anion/water molecules. The location of first minima of $g_{ij}(r)$ is considered as the size of coordination shell and j molecules within this region is considered as co-ordination number (C_n^{i-j}). The cumulative coordination number profile, $C_n^{i-j}(r)$, was obtained by integrating PCF as,

$$C_n^{i-j}(r) = \int_0^r \rho_j \, g_{ij}(r) \, 4\pi r^2 \, dr \, . \tag{7}$$

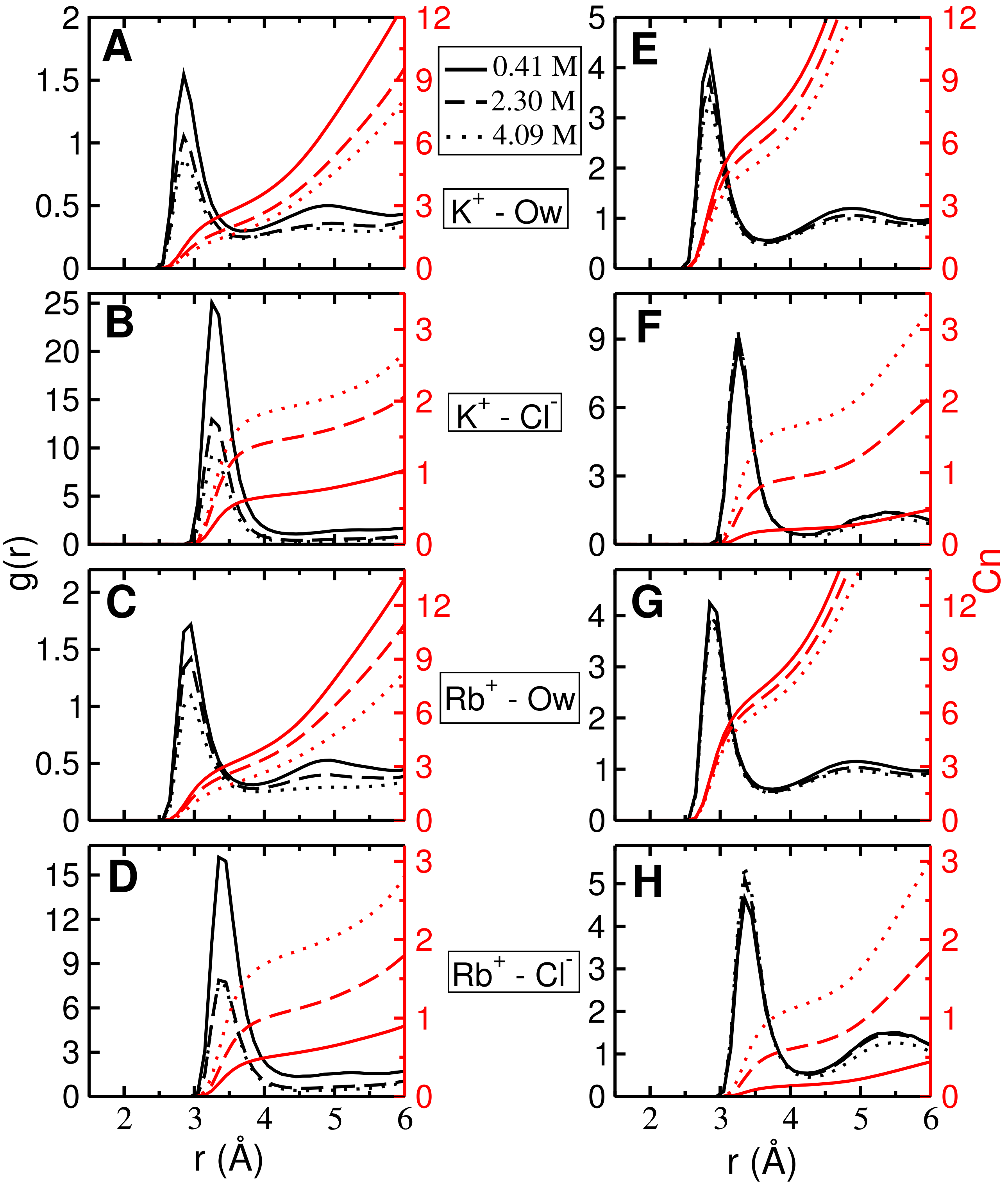

Figure S5. PCF (left *y*-axis) and cumulative coordination number profile (right *y*-axis) of (*A* and *E*) K^+ - Water (oxygen atom), (*B* and *F*) K^+ - Cl^-, (*C* and *G*) Rb^+ - water and (*D* and *H*) Rb^+ - Cl^- pair at various $C_{Rb,B}$ values. The g(r) and $C_n(r)$ profile were evaluated in (A-D, left column) AR and (E-H, right column) bulk region. The location of first minimum of PCF remain constant in both AR and bulk region, however the peak intensity and hence C_n value changes due to different volumes available.

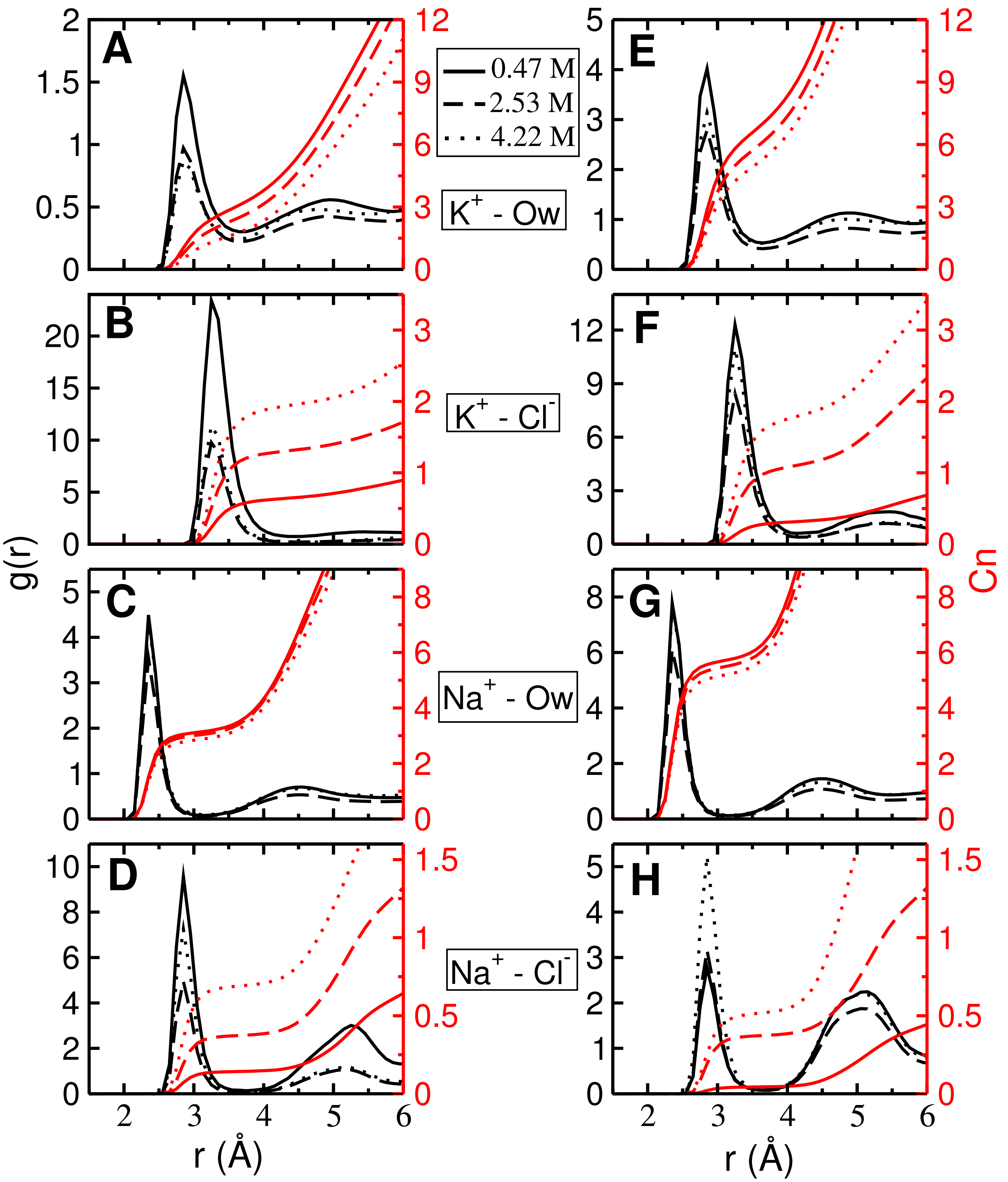

Figure S6. PCF (left *y*-axis) and cumulative coordination number profile (right *y*-axis) of (*A* and *E*) K^+ - Water (oxygen atom), (*B* and *F*) K^+ - Cl^-, (*C* and *G*) Na^+ - water and (*D* and *H*) Na^+ - Cl^- pair at various $C_{Na,B}$ values evaluated in (A-D, left column) AR and (E-H, right column) bulk region. Legends are same as Fig. S5.

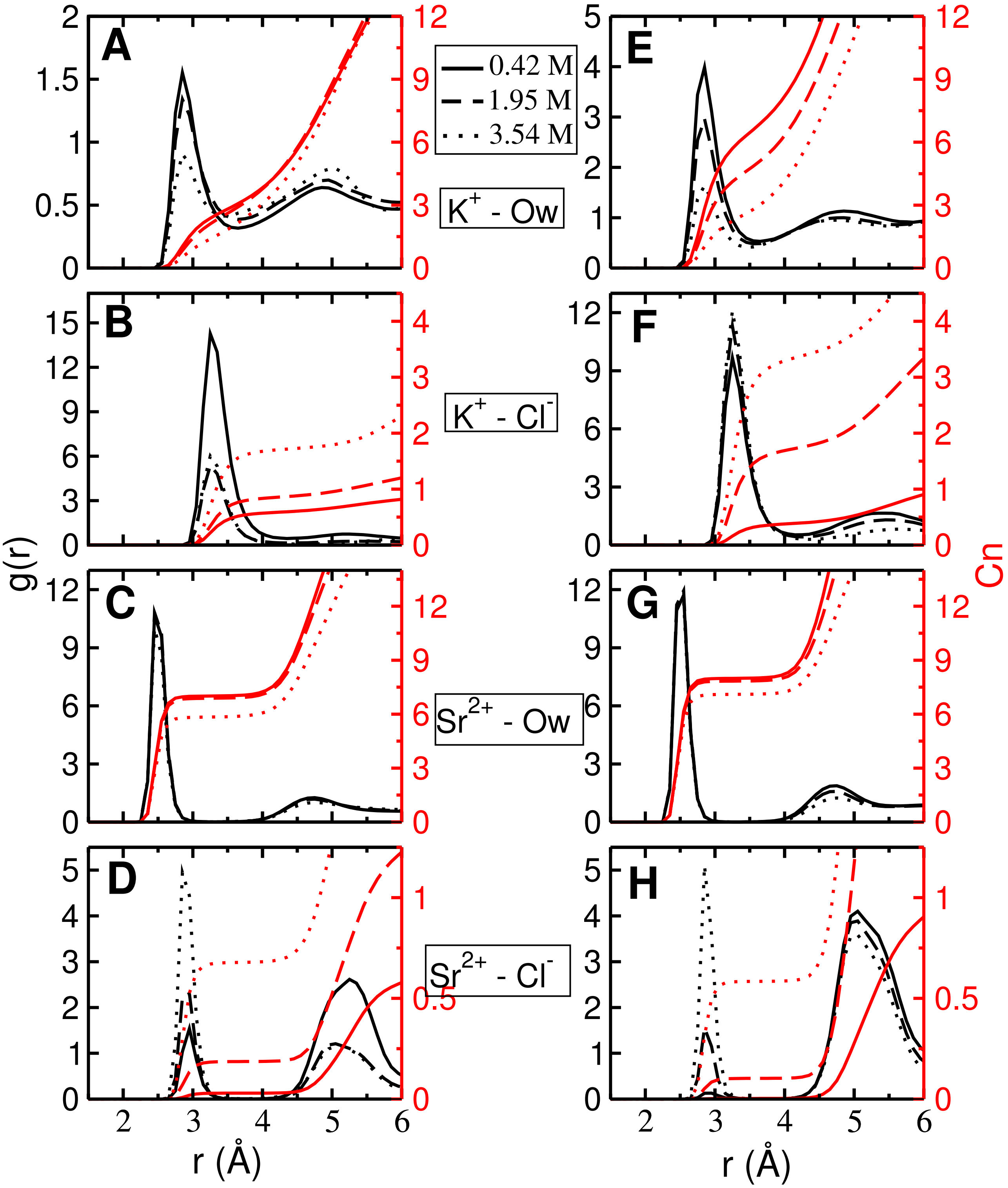

Figure S7. PCF (left *y*-axis) and cumulative coordination number profile (right *y*-axis) of (*A* and *E*) K^+ - Water (oxygen atom), (*B* and *F*) K^+ - Cl^-, (*C* and *G*) Sr^{2+} - water and (*D* and *H*) Sr^{2+} - Cl^- pair at various $C_{Sr,B}$ values evaluated in (A-D, left column) AR and (E-H, right column) bulk region. Legends are same as Fig. S5.

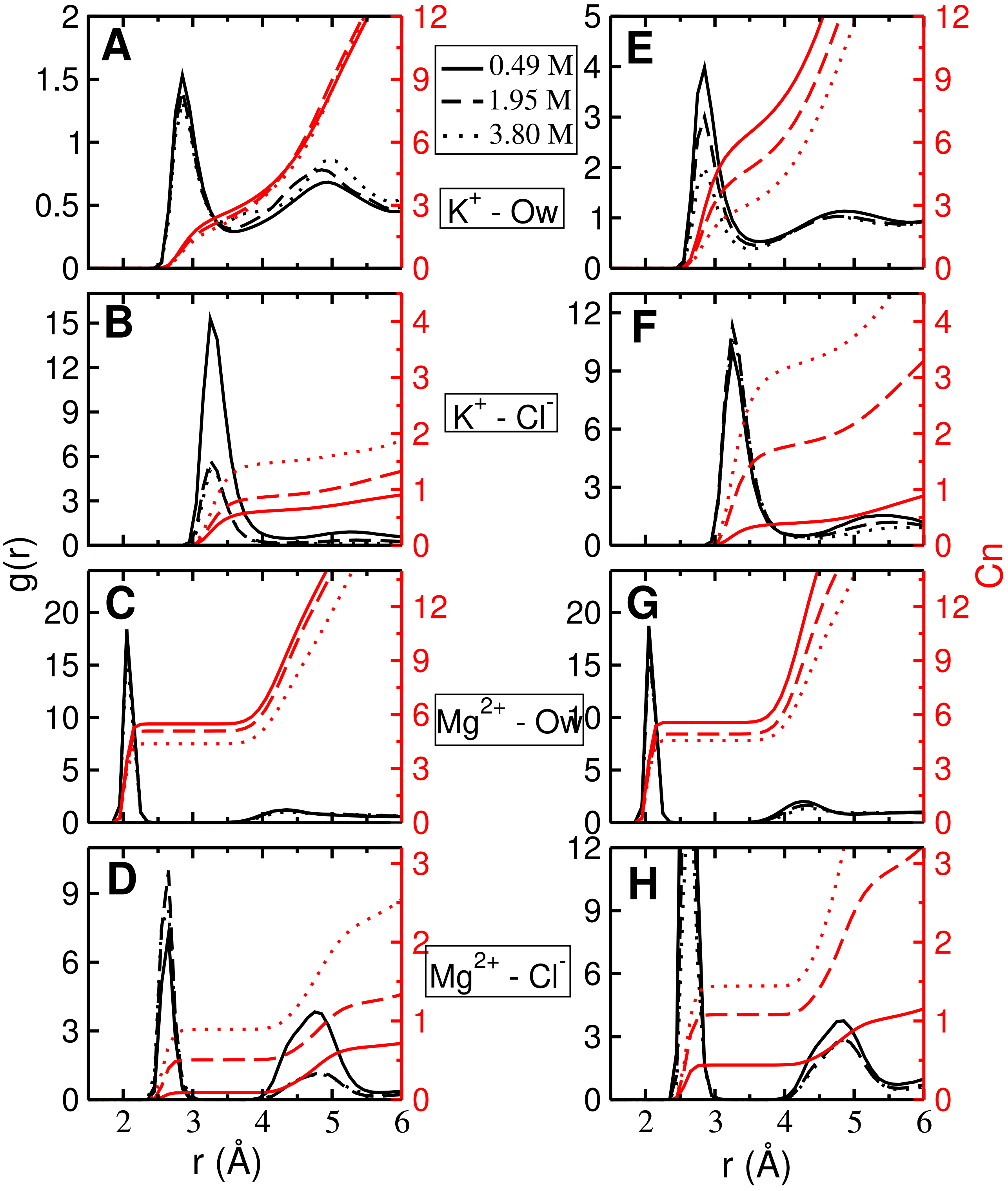

Figure S8. PCF (left y-axis) and cumulative coordination number profile (right y-axis) of (A and E) K^+ - Water (oxygen atom), (B and F) K^+ - Cl^-, (C and G) Mg^{2+} - water and (D and H) Mg^{2+} - Cl^- pair at various $C_{Mg,B}$ values evaluated in (A-D, left column) AR and (E-H, right column) bulk region. Legends are same as Fig. S5.

Table S1. Water molecules hydrating the cations and free water molecules present in the adsorption region (AR) of muscovite mica - RbCl system

	Water molecules of L1 layer					Water molecules of L2 + L3 layers				
$C_{Rb,B}$ (M)	Rb - Ow	K - Ow	(Rb + K) - Ow	Free	Total	Rb - Ow	K - Ow	(Rb + K) - Ow	Free	Total
0.04	0.03	0.24	0.27	28.35	28.62	40.57	139.07	179.64	64.65	244.28
0.10	0.25	0.36	0.60	23.56	24.16	61.05	120.88	181.93	46.85	228.78
0.23	0.23	0.08	0.30	20.53	20.83	77.73	105.81	183.54	42.41	225.95
0.41	0.11	0.09	0.19	17.12	17.31	77.63	101.22	178.85	33.55	212.40
0.52	0.30	0.11	0.41	16.41	16.82	91.15	87.55	178.70	28.29	206.99
0.70	0.24	0.22	0.46	14.43	14.89	92.01	83.23	175.25	24.60	199.85
0.85	0.21	0.13	0.33	13.18	13.51	94.74	76.61	171.35	21.06	192.41
1.01	0.30	0.06	0.36	12.36	12.72	100.95	70.70	171.65	21.62	193.27
1.20	0.24	0.09	0.33	12.49	12.82	99.80	66.34	166.14	19.58	185.72
1.48	0.11	0.01	0.12	7.82	7.94	109.46	49.14	158.60	13.09	171.69
1.95	0.34	0.08	0.41	13.23	13.64	112.06	55.62	167.68	19.92	187.59
2.30	0.28	0.05	0.33	8.85	9.18	114.86	41.62	156.48	14.36	170.85
2.85	0.19	0.09	0.28	4.85	5.13	101.23	42.39	143.62	9.29	152.91
3.48	0.26	0.07	0.33	5.67	6.00	101.81	35.42	137.23	8.99	146.22
3.83	0.44	0.02	0.46	5.67	6.13	107.29	30.98	138.27	8.90	147.17
4.09	0.38	0.00	0.38	2.84	3.22	103.47	21.78	125.25	5.82	131.07
4.62	0.18	0.02	0.20	1.01	1.21	87.45	9.02	96.47	3.03	99.50

$C_{Rb,B}$ - bulk concentration of RbCl salt solution. Second column onwards, M-Ow, list number of water molecules hydrating M-ion. (Rb+K) - Ow list total number of water molecules hydrating Rb^+ and K^+ ion. Free - refers to non-hydrating water molecules.

Table S2. Water molecules hydrating the cations and free water molecules present in the adsorption region (AR) of muscovite mica - NaCl aqueous system (Legends same as Table S1)

	Water molecules of L1 layer					Water molecules of L2 + L3 layers				
$C_{Na,B}$ (M)	Na - Ow	K - Ow	(Na + K) - Ow	Free	Total	Na - Ow	K - Ow	(Na + K) - Ow	Free	Total
0.03	1.93	0.29	2.22	28.04	30.26	33.29	141.62	174.91	76.20	251.10
0.24	2.16	0.57	2.73	27.74	30.47	39.62	136.20	175.83	66.22	242.04
0.31	3.62	0.28	3.90	24.83	28.73	58.13	118.78	176.91	64.29	241.20
0.47	4.52	0.23	4.75	20.65	25.40	67.78	106.15	173.93	58.88	232.80
0.55	5.76	0.33	6.09	21.73	27.82	83.51	93.08	176.58	60.44	237.03
0.81	6.07	0.38	6.45	19.08	25.53	79.66	94.13	173.79	54.82	228.61
0.96	6.54	0.44	6.98	18.54	25.52	86.32	86.05	172.37	53.14	225.52
1.14	7.67	0.40	8.07	19.14	27.21	94.29	77.16	171.45	60.92	232.37
1.30	7.08	0.17	7.25	18.87	26.12	99.15	75.15	174.30	49.93	224.23
1.52	7.24	0.31	7.55	17.13	24.68	98.22	72.91	171.13	48.67	219.80
1.67	8.99	0.39	9.38	16.68	26.06	106.52	65.76	172.28	50.45	222.74
1.90	6.64	0.60	7.24	16.68	23.92	96.62	70.82	167.44	42.80	210.23
2.16	9.30	0.41	9.71	16.95	26.66	111.96	60.79	172.75	52.37	225.11
2.38	8.42	0.19	8.61	16.04	24.65	103.17	69.26	172.43	44.54	216.96
2.54	8.95	0.26	9.21	16.19	25.40	120.03	50.64	170.67	50.15	220.82
2.73	11.59	0.26	11.85	15.08	26.93	118.44	50.98	169.43	46.80	216.23
2.99	10.00	0.25	10.25	14.09	24.34	111.28	56.10	167.38	43.23	210.60
3.13	10.16	0.36	10.51	12.94	23.45	115.95	50.07	166.02	38.79	204.81
3.34	10.11	0.40	10.51	13.55	24.06	119.79	41.59	161.39	44.84	206.22
3.49	10.06	0.45	10.51	11.34	21.84	122.96	38.73	161.69	41.97	203.66
3.84	10.09	0.20	10.29	12.91	23.19	115.06	46.33	161.38	42.33	203.71
4.00	8.71	1.20	9.91	10.79	20.70	114.79	41.77	156.56	38.42	194.98
4.22	11.64	0.31	11.95	10.64	22.59	127.63	30.85	158.48	40.99	199.48
4.44	13.55	0.04	13.59	10.42	24.01	124.65	33.23	157.88	37.09	194.96
4.59	14.54	0.56	15.10	12.74	27.84	137.66	23.34	161.00	42.18	203.18
4.87	12.39	0.22	12.61	10.68	23.29	131.44	30.77	162.21	39.64	201.85

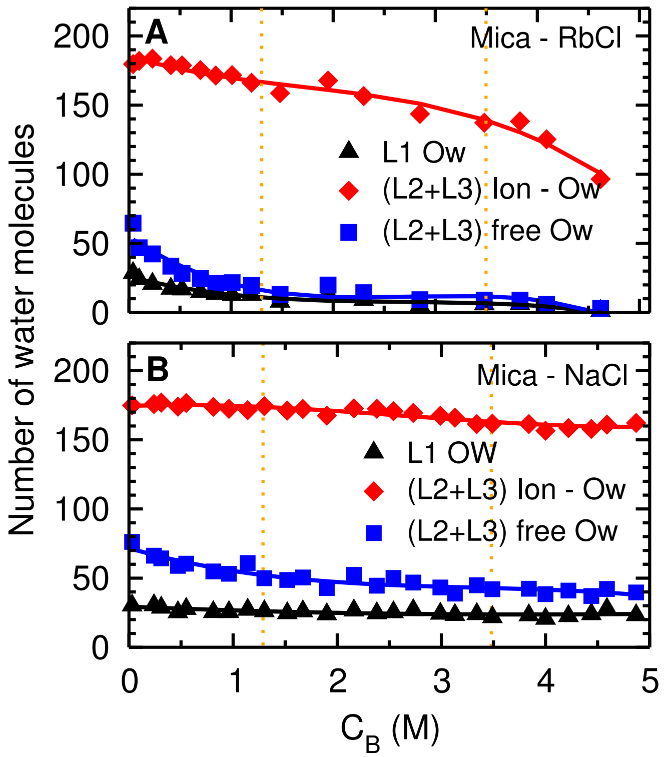

Figure S9. Number of water molecules in L1 layer (L1 Ow - ▲), L2+L3 layer water molecules hydrating cations (⧫) and L2+L3 layer non-hydrating (free) water molecules (■) in (*A*) mica-RbCl and (*B*) mica-NaCl system at various concentration of salt solution C_B.

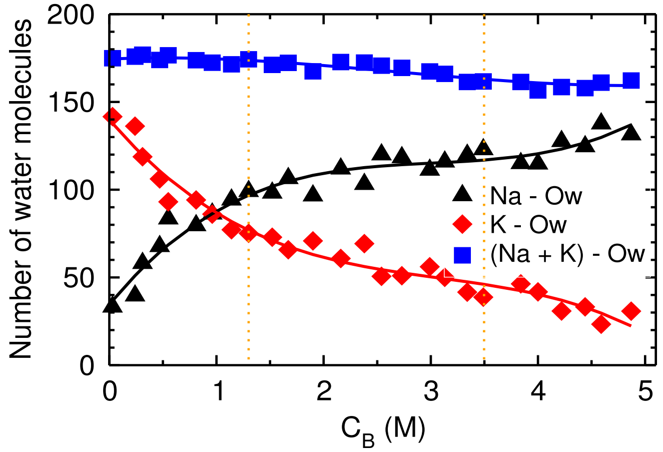

Figure S10. Number of water molecules from L2+L3 layer hydrating the cations (Na^+ - ▲, K^+ - ⧫) at various C_B of NaCl aqueous solution. Redistribution of water molecules from K^+ hydration shell to Na^+ hydration shell is observed giving rise to constant total number of hydrating water molecules (■).

S4. Two-dimensional (2D) density distribution near mica surface

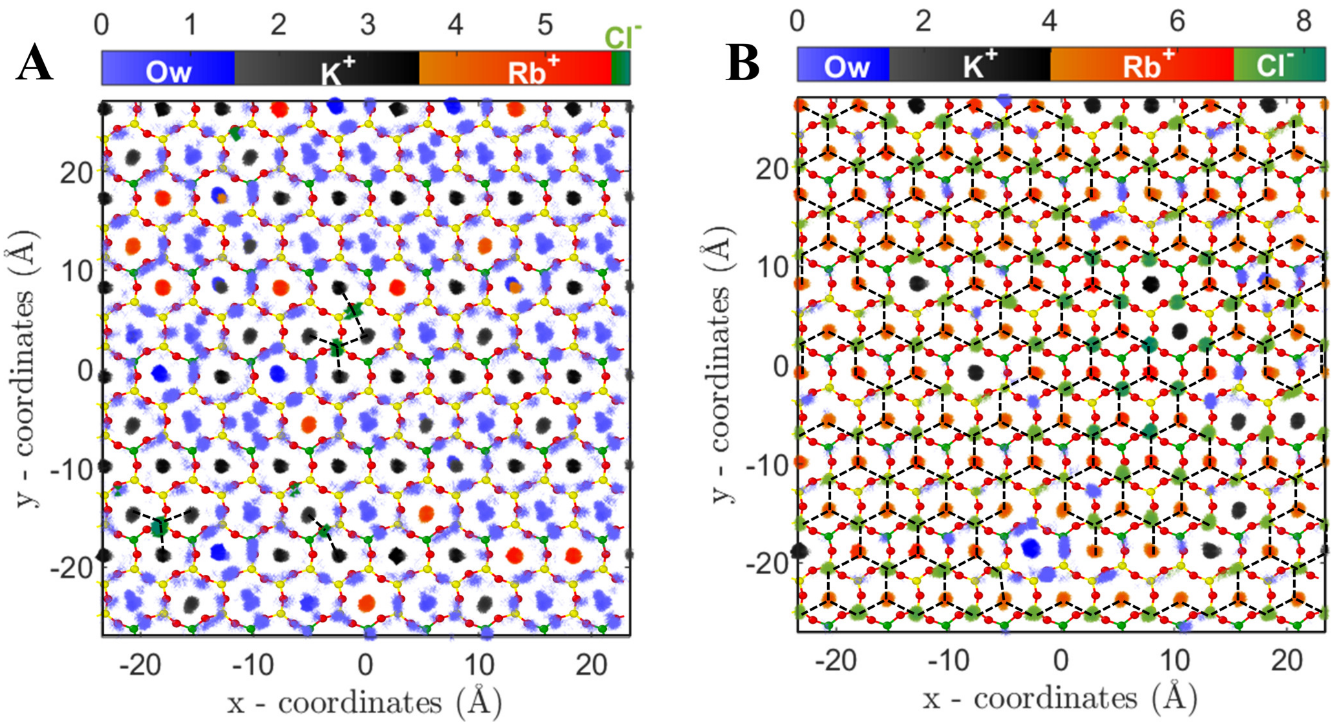

Figure S11. Two-dimensional (2D) density distribution of water, K^+, Rb^+ and Cl^- ions present within AR (in $Å^{-3}$) at (*A*) $C_{Rb,B} = 0.04$ M and (*B*) $C_{Rb,B} = 4.62$ M of RbCl solution shown using separate colorbars.Background is the top view of 001 plane mica surface consisting of silicon(yellow), aluminum(green) and basal oxygen atoms(red). The adsorbing Rb^+ cation, surface K^+ ion and L1 water molecules are adsorbed on the ditrigonal cavities and Cl^- ion adsorb on top of silicon (Si) atom of mica surface. The water molecules present in the L2+L3 layers are spread around the ions on the edges of the cavities. At $C_{Rb,B} = 0.04$ M, few K^+ ions are surrounded by Cl^- ions forming KCl cluster, shown using black dash line, and no RbCl clusters are observed. At $C_{Rb,B} = 4.62$ M, both Rb^+ and K^+ ions are surrounded by Cl^- ions forming RbCl and KCl cluster. The Rb^+ and Cl^- atoms form a giant cluster of size around 105, shown by black dash lines, spanning the entire surface confirming the salting out phenomena. At this higher $C_{Rb,B}$ values, very few pockets of interfacial water molecules are observed within AR.

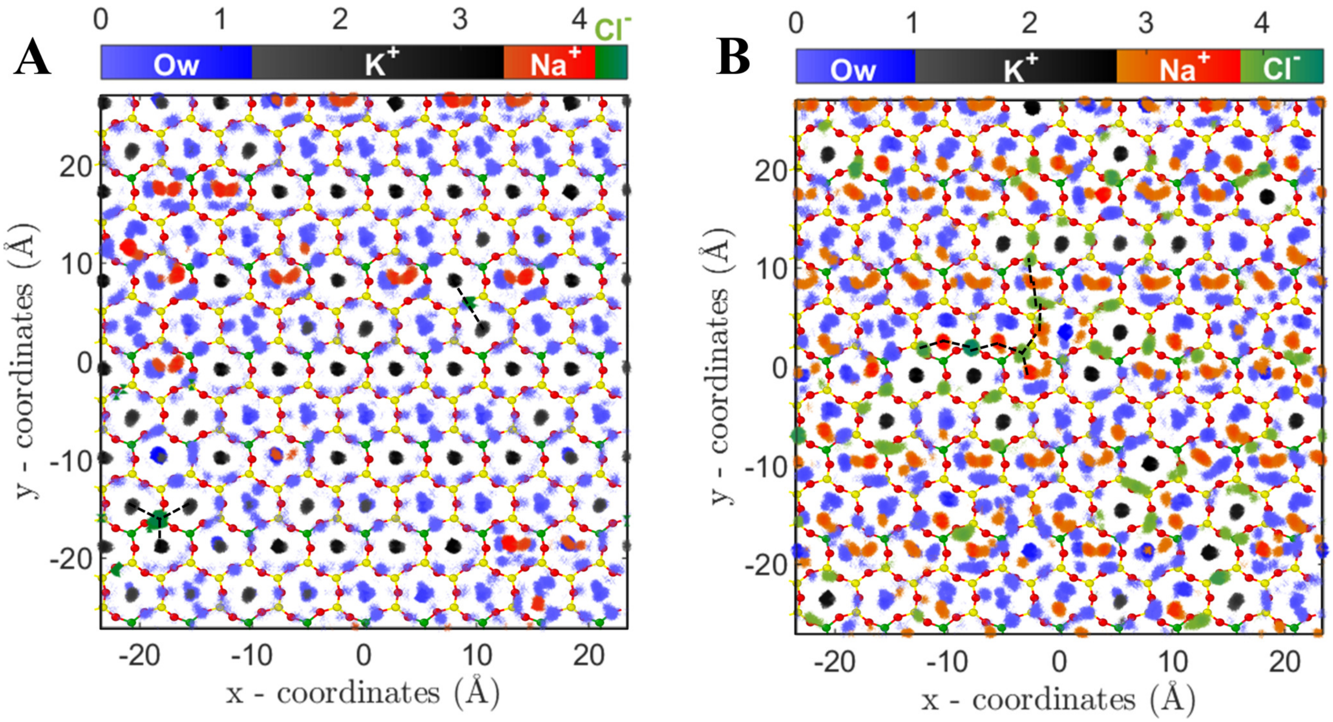

Figure S12. 2D density distribution of water, K^+, Na^+ and Cl^- ions present within AR (in $Å^{-3}$) at (*A*) $C_{Na,B} = 0.03$ M (*B*) $C_{Na,B} = 4.87$ M of NaCl solution shown using separate colorbars. Remaining legends are same as Fig. S11. At $C_{Na,B} = 0.03$ M, few K^+ ions are surrounded by Cl^- ions forming KCl cluster size of 4 and 3 (shown by dotted lines). At $C_{Na,B} = 4.87$ M, both Na^+ and K^+ ions are surrounded by Cl^- ions forming NaCl and KCl cluster.

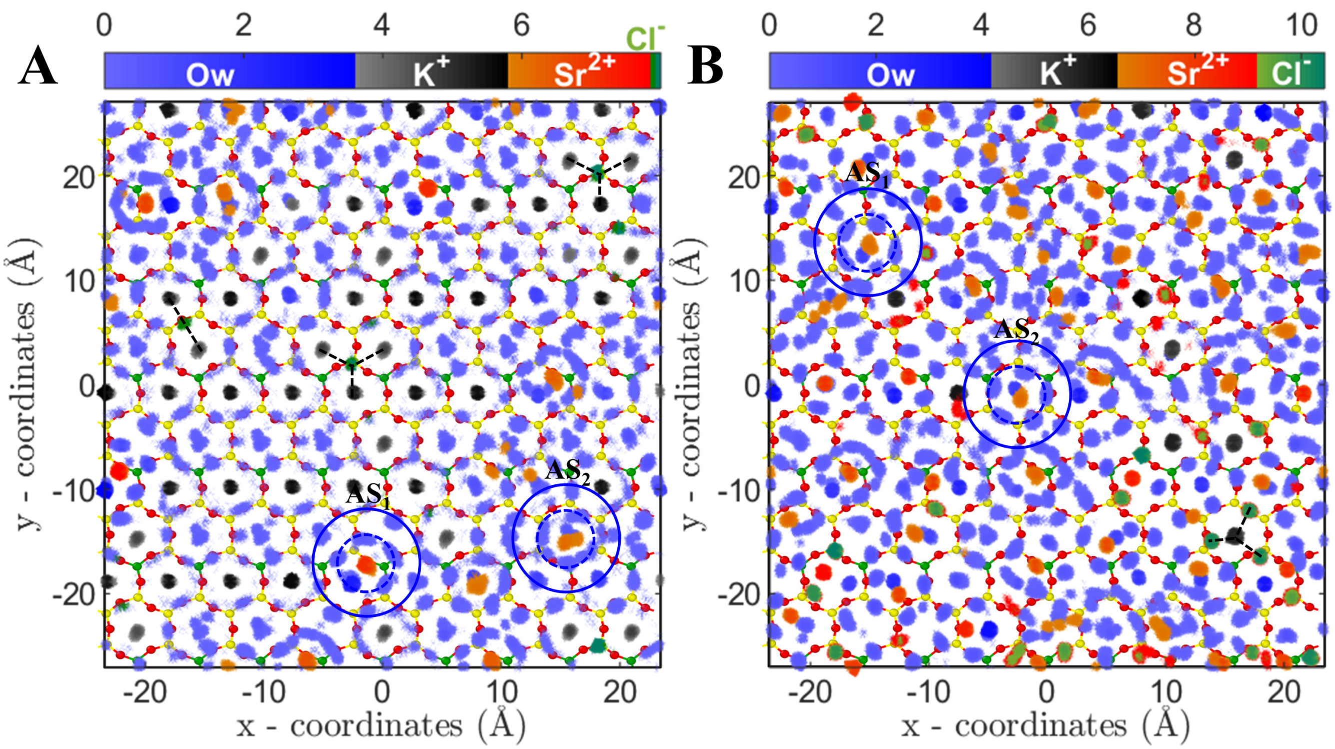

Figure S13. 2D density distribution of water, K^+, Sr^{2+} and Cl^- ions present within AR (in $Å^{-3}$) at (*A*) $C_B = 0.42$ M and (*B*) $C_B = 3.54$ M of $SrCl_2$ solution shown using separate color bars. Legends are same as Fig. S11. Sr^{2+} ions adsorb near bridging oxygen atoms (AS_1) as well as on the ditrigonal cavities (AS_2). Water molecules form a hydration shell around divalent ions by adsorbing in nearby cavities; first and second hydration shell are highlighted by blue dotted and continuous circle. These hydration water molecules prevents adsorption of other cations in the nearby cavities via steric repulsion. This leads to lesser adsorption and early saturation of adsorption isotherm of divalent ions. Few K^+ ions are surrounded by Cl^- ions forming KCl clusters (shown by dotted lines) where as Sr^{2+} ions forms cluster either connected by the Cl^- ions present in its hydration shell or by Cl^- ions present beyond AR, just after the 5 Å.

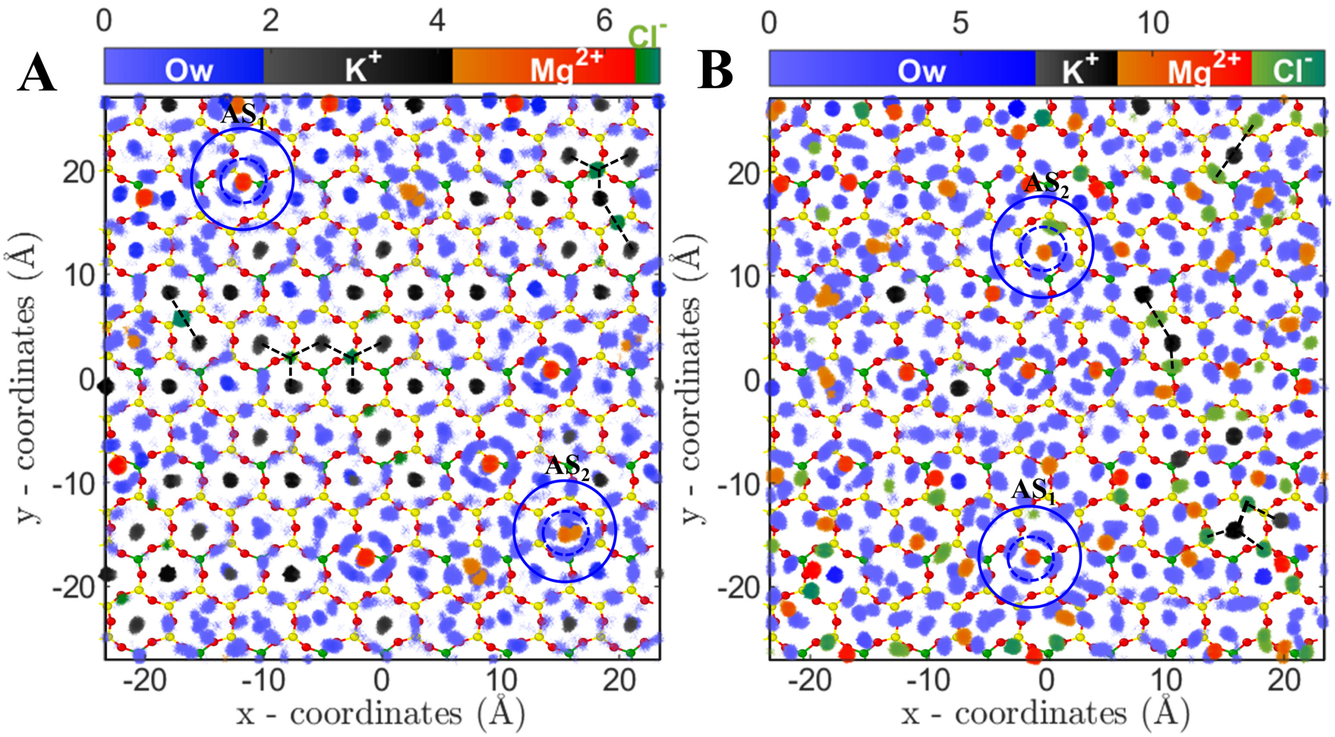

Figure S14. 2D density distribution of water, K^+, Mg^{2+} and Cl^- ions present within AR (in $Å^{-3}$) at (*A*) $C_{Mg,B} = 0.49$ M and (*B*) $C_{Mg,B} = 3.80$ M of $MgCl_2$ solution shown using separate color bars. Legends are same as Fig. SS13.

S5. Cluster size analysis of divalent ions

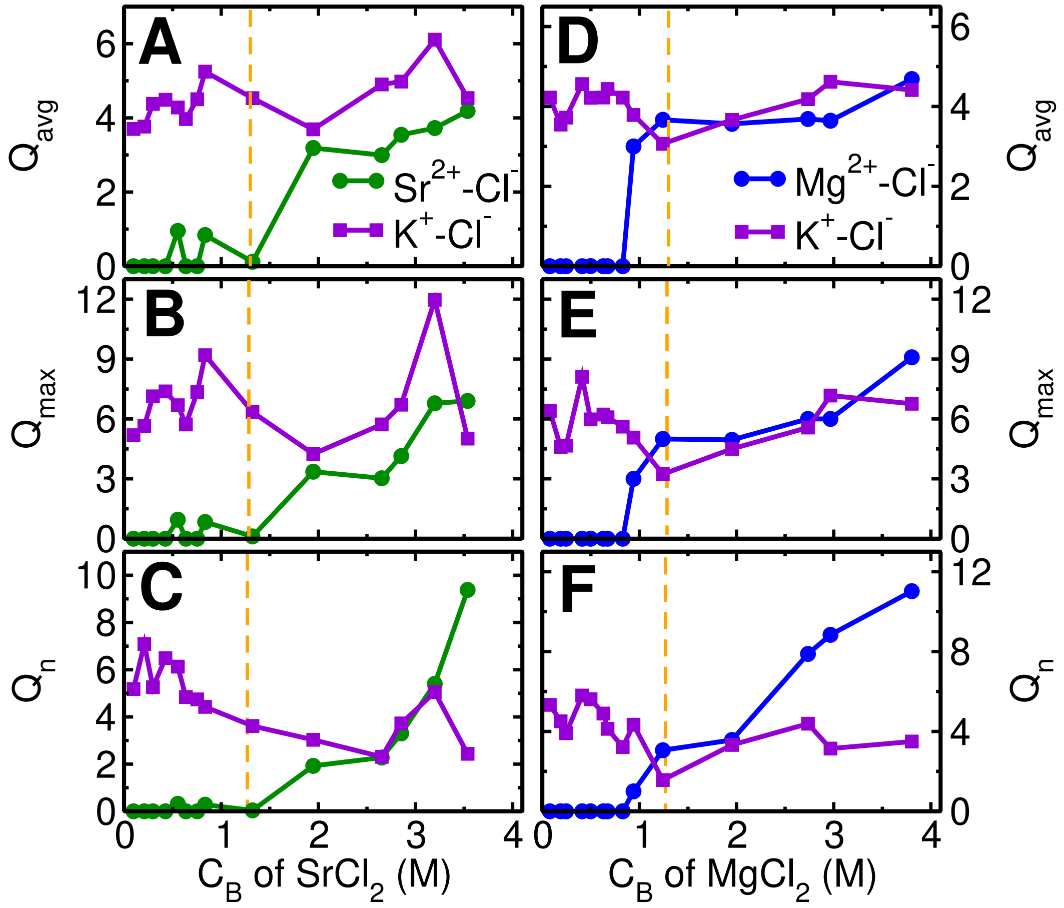

Figure S15. (*A* and *D*) Average cluster size (Q_{avg}), (*B* and *E*) Maximum cluster size (Q_{max}) and (*C* and *F*) Number of clusters (Q_n) formed in the adsorption region at various C_B of $SrCl_2$ and $MgCl_2$ aqueous systems, respectively. The vertical dotted lines are used to indicate various adsorption isotherm regimes. Tightly binding hydration shell of divalent ions restrict their cluster formation with Cl^- anions. Whereas, Cl^- association with K^+ ions lead to formation of KCl clusters. Only at higher C_B, that divalent cation-anion (SrCl and MgCl) clusters are formed.

Table S3. Potential parameters used in the simulation

Molecules	Force-field	Atoms	ε (kJ mol^{-1})	σ (Å)	q (e)
Water	SPC/E[a]	O	0.6502	3.166	- 0.8476
		H	0.0	0.0	+0.4238
Mica	CLAYFF[b]	O1 (bridging oxygen of tetrahedral layer)	0.6502	3.166	-1.05
		O2 (bridging oxygen of octahedral layer)	0.6502	3.166	-1.16875
		O (Hydroxyl)	0.6502	3.166	- 0.95
		H (Hydroxyl)	0.0	0.0	+0.425
		Si	7.007 x 10^{-6}	3.302	+2.1
		Al (substituted Al of tetrahedral layer)	7.007 x 10^{-6}	3.302	+1.575
		Al (of octahedral layer of mica)	7.007 x 10^{-6}	3.302	+1.575
		K^+	0.4184	3.334	+1
Ions	SD[c]	Na^+	0.5447	2.35	+1
	JC[d]	Rb^+	1.8623	3.0949	+1
	Aq[e]	Mg^{2+}	3.6634	1.6444	+2
	Ma[f]	Sr^{2+}	0.25	3.10	+2
	SD[c]	Cl^-	0.41868	4.45	-1

[a] Berendsen et al.[6]; [b] Cygan et al.[7]; [c] Smith and Dang[8]; [d] Joung and Cheatham[9]
[e] Aaquist[10]; [f] Mamatkulov et al.[11]

References

1. Patey, G. N. & Valleau, J. P. A Monte Carlo method for obtaining the interionic potential of mean force in ionic solution. *J. Chem. Phys.* **63**, 2334–2339 (1975).
2. Frenkel, D. & Smit, B. *Understanding Molecular Simulation: From Algorithms to Applications* (Academic Press, 1996).
3. Kumar, S., Rosenberg, J. M., Bouzida, D., Swendsen, R. H. & Kollman, P. A. The weighted histogram analysis method for free-energy calculations on biomolecules. I. The method. *J. Comput. Chem.* **13**, 1011–1021 (1992).
4. Plimpton, S. Fast Parallel Algorithms for Short-Range Molecular Dynamics. *J. Comput. Phys.* **117**, 1–19 (1995).
5. Kobayashi, K. *et al.* Ion Distribution and Hydration Structure in the Stern Layer on Muscovite Surface. *Langmuir* **33**, 3892–3899 (2017).
6. Berendsen, H. J. C., Grigera, J. R. & Straatsma, T. P. The missing term in effective pair potentials. *J. Phys. Chem.* **91**, 6269–6271 (1987).
7. Cygan, R. T., Liang, J.-J. & Kalinichev, A. G. Molecular Models of Hydroxide, Oxyhydroxide, and Clay Phases and the Development of a General Force Field. *J. Phys. Chem. B* **108**, 1255–1266 (2004).
8. Smith, D. E. & Dang, L. X. Computer simulations of NaCl association in polarizable water. *J. Chem. Phys.* **100**, 3757 (1994).
9. Joung, I. S. & Cheatham, T. E. Determination of alkali and halide monovalent ion parameters for use in explicitly solvated biomolecular simulations. *J. Phys. Chem. B* **112**, 9020–9041 (2008).
10. Aaqvist, J. Ion-water interaction potentials derived from free energy perturbation simulations. *J. Phys. Chem.* 8021–8024 (1990).
11. Mamatkulov, S., Fyta, M. & Netz, R. R. Force fields for divalent cations based on single-ion and ion-pair properties. *J. Chem. Phys.* **138** (2013).
